# OBSCN undergoes extensive alternative splicing during human cardiac and skeletal muscle development

**DOI:** 10.1186/s13395-025-00374-6

**Published:** 2025-03-01

**Authors:** Ali Oghabian, Per Harald Jonson, Swethaa Natraj Gayathri, Mridul Johari, Ella Nippala, David Gomez Andres, Francina Munell, Jessica Camacho Soriano, Maria Angeles Sanchez Duran, Juha Sinisalo, Heli Tolppanen, Johanna Tolva, Peter Hackman, Marco Savarese, Bjarne Udd

**Affiliations:** 1 Folkhälsan Research Center, Helsinki, Finland; 2 Research Program for Clinical and Molecular Metabolism, Faculty of Medicine, University of Helsinki, 00014 Helsinki, Finland; 3 Department of Medical Genetics, University of Helsinki, Helsinki, Finland; 4 Harry Perkins Institute of Medical Research, Centre for Medical Research, University of Western Australia, Nedlands, WA Australia; 5 Pediatric Neuromuscular Unit. Child Neurology Department. Hospital Universitari Vall d’Hebron, Vall d’Hebron Research Institute (VHIR) ES, Barcelona, Spain; 6 Histology Department, Vall d’Hebron University Hospital ES, Barcelona, Spain; 7 Prenatal Diagnosis Department, Vall D’hebron University Hospital ES, Barcelona, Spain; 8 Department of Obstetrics, Maternal Fetal Medicine Unit, Universitat Autònoma de Barcelona, Hospital Vall D’hebron, Barcelona, Spain; 9 Helsinki University Central Hospital, Helsinki, Finland; 10 Department of Pathology, Transplantation Laboratory, University of Helsinki, Helsinki, Finland; 11 Department of Neurology, Neuromuscular Research Center, Tampere University and University Hospital, Tampere, Finland

**Keywords:** Neuromuscular diseases, OBSCN/RNA splicing, Exon inclusion, Muscle development

## Abstract

**Background:**

Highly expressed in skeletal muscles, the gene Obscurin (*i.e. OBSCN)* has 121 non-overlapping exons and codes for some of the largest known mRNAs in the human genome. Furthermore, it plays an essential role in muscle development and function. Mutations in *OBSCN* are associated with several hypertrophic cardiomyopathies and muscular disorders. *OBSCN* undergoes extensive and complex alternative splicing, which is the main reason that its splicing regulation associated with skeletal and cardiac muscle development has not previously been thoroughly studied.

**Methods:**

We analyzed RNA-Seq data from skeletal and cardiac muscles extracted from 44 postnatal individuals and six fetuses. We applied the intron/exon level splicing analysis software IntEREst to study the splicing of *OBSCN* in the studied samples. The differential splicing analysis was adjusted for batch effects. Our comparisons revealed the splicing variations in *OBSCN* between the human skeletal and cardiac muscle, as well as between post-natal muscle (skeletal and cardiac) and the pre-natal equivalent muscle.

**Results:**

We detected several splicing regulations located in the 5’end, 3’ end, and the middle of *OBSCN* that are associated with human cardiac or skeletal muscle development. Many of these alternative splicing events have not previously been reported. Our results also suggest that many of these muscle-development associated splicing events may be regulated by *BUB3*.

**Conclusions:**

We conclude that the splicing of *OBSCN* is extensively regulated during the human skeletal/cardiac muscle development. We developed an interactive visualization tool that can be used by clinicians and researchers to study the inclusion of specific OBSCN exons in pre- and postnatal cardiac and skeletal muscles and access the statistics for the differential inclusion of the exons across the studied sample groups. The *OBSCN* exon inclusion map related to the human cardiac and skeletal muscle development is available at http://psivis.it.helsinki.fi:3838/OBSCN_PSIVIS/. These findings are essential for an accurate pre- and postnatal clinical interpretation of the *OBSCN* exonic variants*.*

**Supplementary Information:**

The online version contains supplementary material available at 10.1186/s13395-025-00374-6.

## Background

**Fig. 1:**
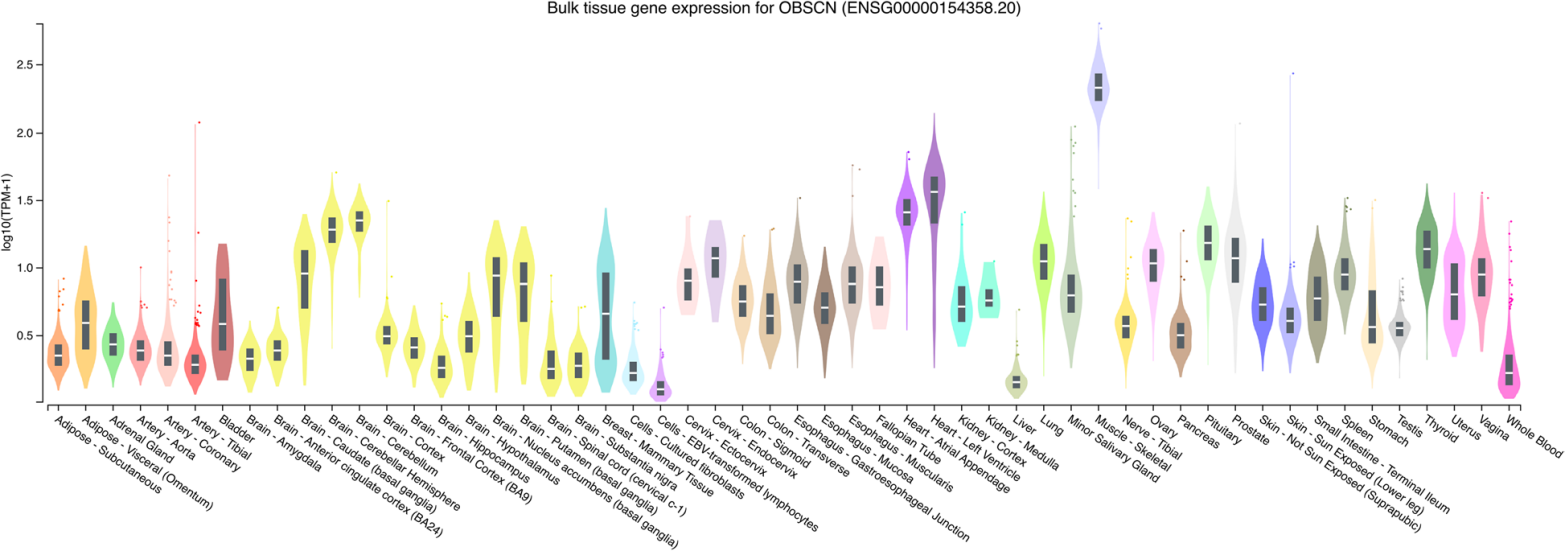
Expression of *OBSCN* in various tissues. Log_10_ scaled expression of *OBSCN* across different tissues available in GTEX Analysis Release V8. The curves show the density of the expression values. The horizontal line within the black box shows the median. The black box plots extend vertically from the 25th to the 75th percentile. The outliers are expression levels higher or lower than 1.5 time the interquartile range. GTEx Portal (Analysis Release V8) on 09/13/23

Discovered about two decades ago, the name of the gene Obscurin (*OBSCN*) refers to the challenges that were endured by the researchers in its initial detection and characterization [1]. These challenges were mainly caused by the large size and the relatively low abundance of the transcripts in most tissues. With 121 non-overlapping exons, *OBSCN* is indeed one of the genes that code for the largest mRNAs in the human genome. In human, it is expressed at highest levels in skeletal muscles, however it is also highly expressed in cardiac muscles (Fig. 1). With the relatively high number of exons, we speculate that *OBSCN* undergoes extensive alternative splicing, especially exon skipping. These events are known to result in different mRNAs that code for a family of proteins characterized as “obscurins” [1, 2]. The two largest *OBSCN* isoforms are obscurin A and obscurin B [3]. They are both characterized by a long stretch of more than 60 immunoglobulin (*i.e.* Ig) repeats that are interrupted by a calmodulin-binding IQ motif and several domains such as fibronectin type-III, SRC homology 3, Rho guanine nucleotide exchange factor and pleckstrin homology. The former (*i.e.*, obscurin A), which is also the smaller of the two (*i.e.* ~ 720 kDa), features a non-modular C-terminal that includes several phosphorylation sites. In contrast, the C-terminus of obscurin B (*i.e.* ~ 870 kDa) features a fibronectin type-III domain, two additional Ig sites and two serine/threonine type kinase sites (for a comprehensive review see Grogan A., et al*.*) [3]. Furthermore, other smaller isoforms are reported to be highly abundant in cardiac muscles. Some of these isoforms solely feature an Ig site, a fibronectin type-III site, and one or two kinase domains [3–5].

Obscurin is associated with neuromuscular functions such as myofibrillogenesis, hypertrophic response and cytoskeletal arrangements [3]. Furthermore, obscurin interacts with the giant sarcomere protein titin [6]. In fact, obscurin/titin disrupting mutations are suggested to be the cause of several hypertrophic cardiomyopathies and muscular disorders [7–9]. Despite the important role of *OBSCN* in the skeletal muscle development and functioning, to our knowledge, the splicing regulation of *OBSCN* has not yet been studied in detail. In this study, we analyzed 75 pre- and post-natal skeletal muscle and heart (*i.e.* 45 postnatal muscles, 7 postnatal hearts, 20 fetal muscles and 3 fetal hearts) RNAseq data. The fetal samples have been denoted with “F”, and the muscle and heart samples are referred to as “M” and “H” respectively. However, to prevent confusion with “prenatal” we have labelled the postnatal samples with “A” as most of these samples are from adults. In particular, we studied the well-known isoforms of *OBSCN* to quantify and compare the inclusion levels of the *OBSCN* exons across the studied samples. This allowed us to characterize the splicing regulation of *OBSCN* in cardiac and skeletal muscle development by comparing pre- and post-natal samples. As the role of obscurin in muscle diseases is an emerging topic of study [8, 10], a detailed map of the splicing and the expression of various isoforms of this gene is of great importance for the correct interpretation of the effects of novel variants.

## Results

The prenatal analysis consisted of analyzing inhouse RNA-Seq data from fetal skeletal muscles (*n* = 20) and fetal cardiac muscles (*n* = 2) from 2 different fetuses (Table 1). Additionally, it included analyzing publicly available skeletal muscle data from a 19 weeks female, skeletal muscle data from a 22 weeks male, cardiac muscle data from a 28 weeks female and cardiac muscle data from a 19 weeks female (for more information see ‘Methods’). For postnatal analysis, RNA-Seq data from an internal cohort of 44 individuals were analyzed (Table 1). We studied six *OBSCN* isoforms, four of which were curated mRNAs and featured NM REFSEQ IDs, *i.e.* ENST00000284548 (NM_052843), ENST00000422127 (NM_001098623), ENST00000570156 (NM_001271223) and ENST00000680850 (NM_001386125). Additionally, we analyzed isoforms ENST00000660857 and ENST00000493977 since they collectively featured four additional unique exons. Overall, we extracted 126 unique or 121 non-overlapping exons from these isoforms, out of which three exons were annotated as alternative first and four exons as alternative last. Additionally, the introns upstream of two exons were annotated with alternative 3’ splicing (Table 2).

**Table 1:** Internal cohort of individuals whose biopsies were collected for RNA-seq

**Sex**	**No. samples**
M	21
F	12
NA	13
**Age at biopsy (years)**	
0–4	4
5–20	7
21–60	25
> 60	5
NA	5
**Clinical conditions**	
Unsolved myopathy	24
Myopathy with a genetic diagnosis	13
Amputees for myopathy-unrelated reasons	4
Hyperckemia without myopathology	5

### Exon inclusion/skipping

**Fig. 2:**
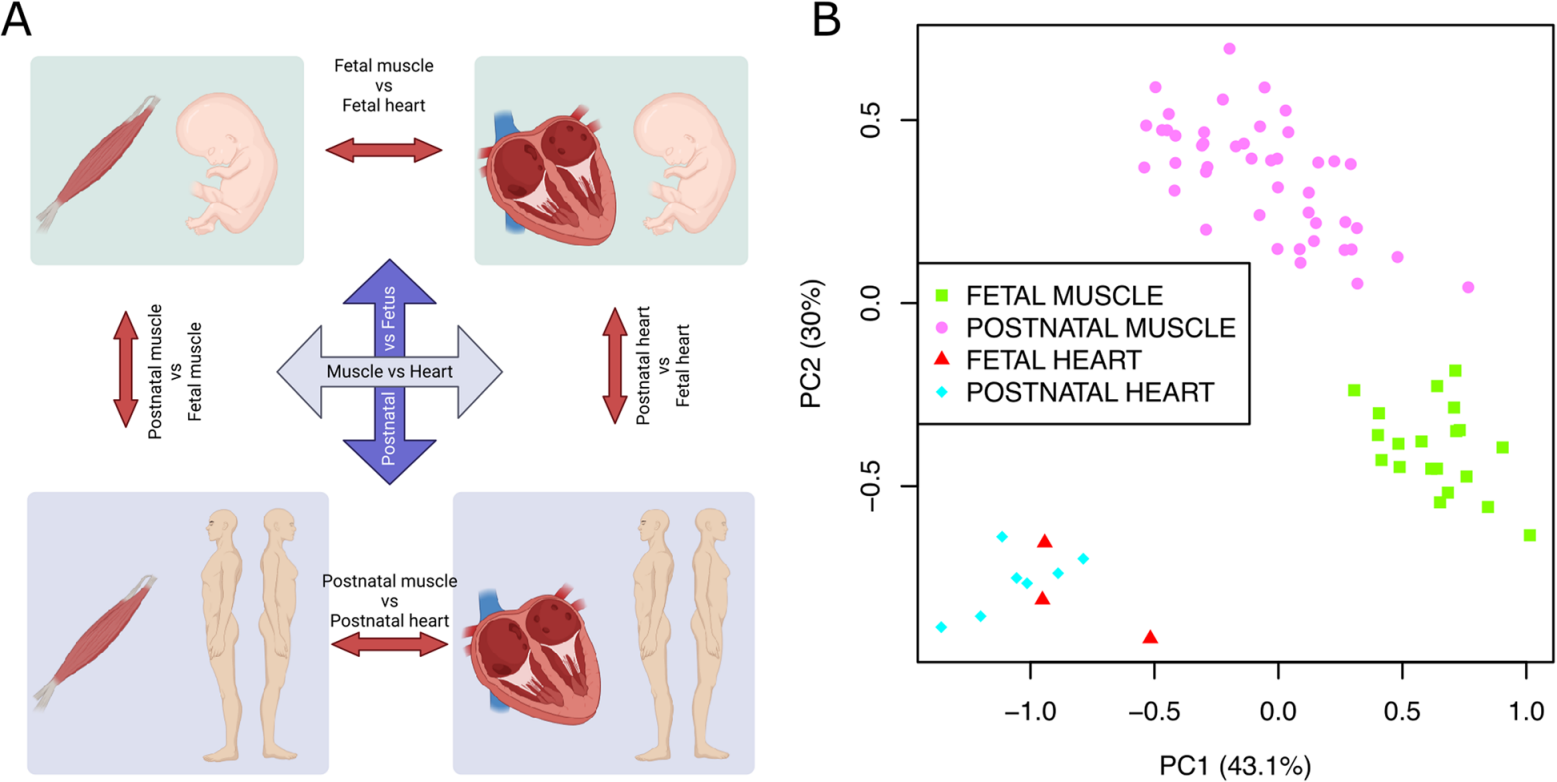
Sample comparisons in our study. **A** The RNA-Seq data in our study were analyzed for splicing by comparing the muscle samples to heart samples (denoted with M), mostly adult postnatal samples to fetal samples (denoted with A), mostly adult postnatal muscle samples to fetal muscle samples (denoted also with AM/FM), mostly adult postnatal heart samples to fetal heart samples (AH/FH), mostly adult postnatal muscle samples to mostly adult postnatal heart samples (AM/AH), and fetal samples to fetal heart samples (FM/FH). **B** Scatterplot shows the separation of the studied samples based on *OBSCN* exon inclusion PSI values, by illustrating PC1 vs PC2 (achieved from PCA analysis). The sample types have been labelled with different shapes and colours

**Fig. 3:**
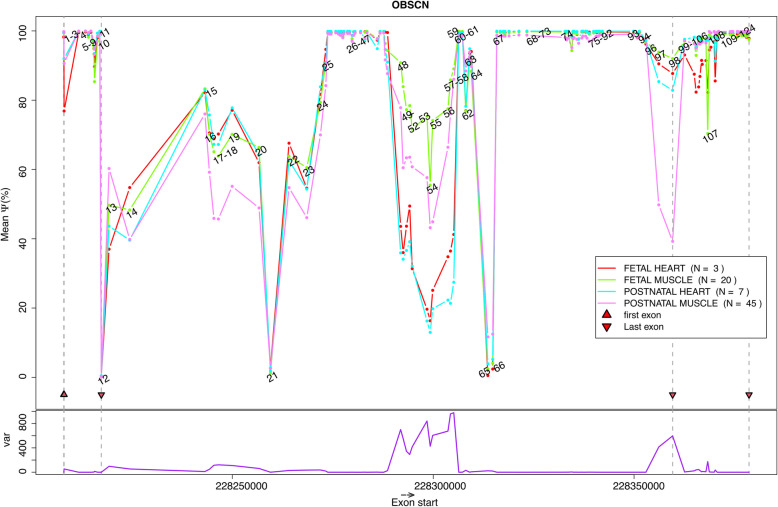
Inclusion levels of *OBSCN* exons: The plot illustrates the average $\Psi$ inclusion levels of the unique exons of *OBSCN* (in the 4 studied sample classes, *i.e.* postnatal muscles, postnatal hearts, fetal muscles and fetal hearts) and the exons are ordered by their start position (*i.e.* X- axis). Each dot shows the average of the $\Psi$ values for an *OBSCN* exon within a sample class. The dots are marked by the exon numbers. For those dots that are too close together to distinguish, the ranges of the exon numbers are stated. The average $\Psi$ measurements related to a sample class are connected via a line. As their $\Psi$ measurements are not accurate (due to the lack of exon-skipping sequence reads), the first and last exons are shown with red triangles and horizontal grey dashed-lines. The variance of the average $\Psi$ values (across the different sample classes) are shown (with a purple line) below, in the figure. Exon 126 (i.e. an alternative last exon) is omitted as its start coordinate is identical to that of exon 125

**Fig. 4:**
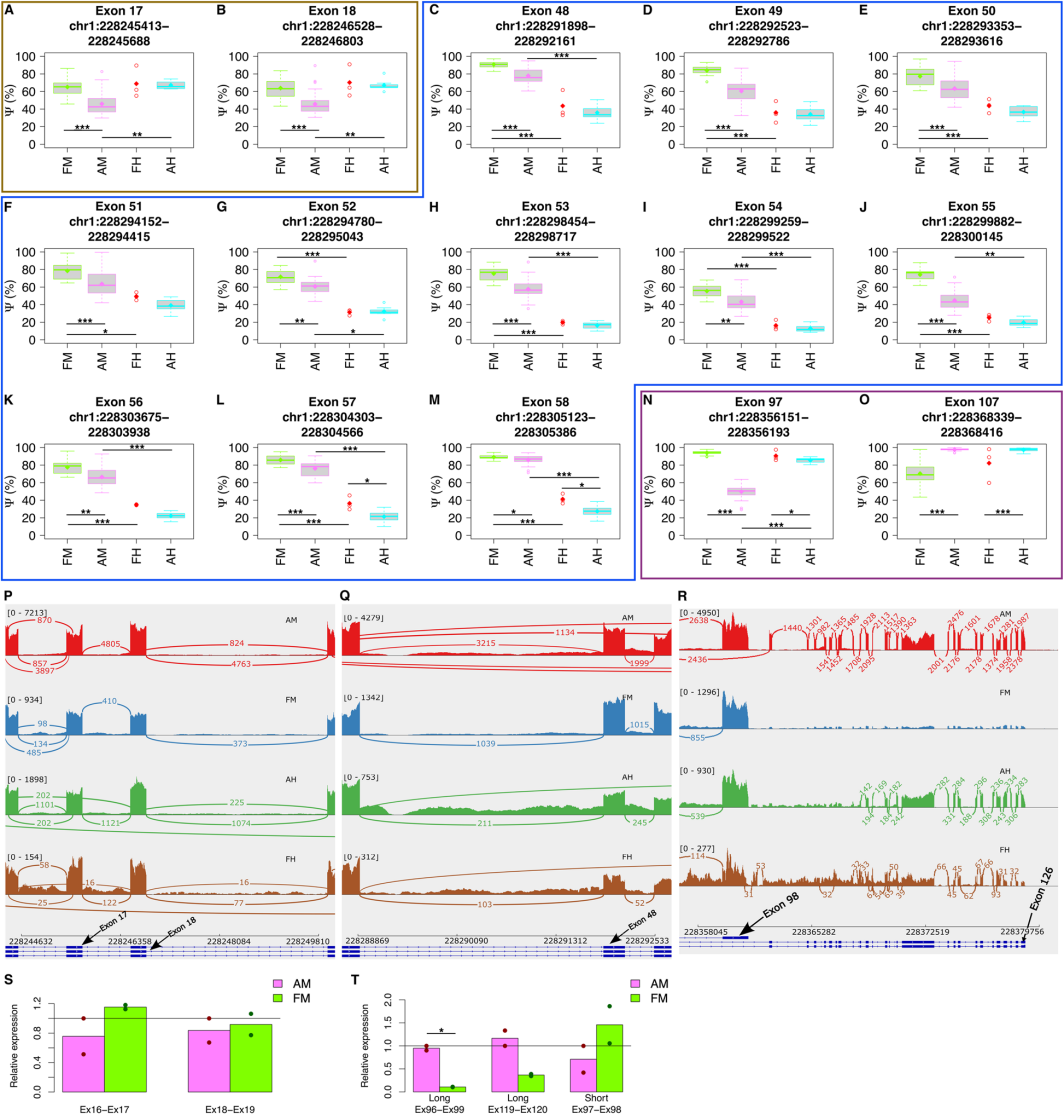
Significantly differentially included *OBSCN* exons: **A**-**O**) Ordered by the exon number, the boxplots illustrate the distribution of the $\Psi$ levels of the exons that were detected as significant (FDR < 0.05) in at least three of the comparisons performed in the study. The box plots extend from the 25th to the 75th percentile, and the thick horizontal line represents the median. The whiskers of the box plots show 1.5 times the interquartile range. The outliers are values higher and lower than the interquartile range. **P**-**R** Sashimi plots, illustrate the exon-exon junctions observed in the RNAseq data, in regions flanking exons 17, 18, 48, 98 and 126. The samples with the nearest PSI values to the median PSI of the exons are chosen for the sashimi plots. **S**-**T** Relative expression levels of the mRNAs that include exons 17 and 18 (S) (based on primers matching junctions Ex16-Ex17 and Ex18-Ex19), and relative expression of the long and short *OBSCN* isoforms (T) (based on analyzing exon-exon junctions specific to these isoforms) are shown with bar plots. These values were measured by real-time polymerase chain reaction (*i.e.* RT-qPCR) in the postnatal and fetal skeletal muscles. The exon-exon junction levels have been normalized to the total OBSCN mRNA levels (*i.e.* inferred by Ex67-Ex68 junction in *S* and Ex5-Ex6 junction in *T*). The sample classes include: mostly adult postnatal muscles (AM), mostly adult postnatal hearts (AH), fetal muscles (FM), and fetal hearts (FH). The significant levels in the plots are shown using asterisks: *P* < 0.05 (*), *P* < 0.01 (**) and *P* < 0.001 (***)

We measured $\Psi$ inclusion levels of the 126 *OBSCN* exons for the 45 postnatal skeletal muscle, seven postnatal heart, 20 fetal skeletal muscle and three fetal heart samples. Heatmap (coupled with hierarchical clustering using the “Euclidean” distance) and PCA analysis of *OBSCN* exon inclusion PSI values showed that the samples did not group based on the sex and clinical diagnosis of the studied individuals (Fig. S1A,B, Additional File 1). However, overall, a clear distinction of pre- and postnatal skeletal muscles with a less distinction of pre- and postnatal cardiac muscles was observed (Fig. 2B, and Fig. S2, Additional File 1). We compared the samples in six different ways: the muscle samples to heart samples (*i.e.* denoted with M), postnatal (i.e. mostly from adult individual) samples to fetal samples (*i.e.* A), postnatal muscle samples to fetal muscle samples (*i.e.* AM/FM), postnatal heart samples to fetal heart samples (*i.e.* AH/FH), postnatal muscle samples to postnatal heart samples (*i.e.* AM/AH), and fetal samples to fetal heart samples (*i.e.* FM/FH) (Fig. 2A). We plotted the average $\Psi$ levels to detect loci within *OBSCN* where differential splicing was detected when fetal samples were compared to postnatal samples or heart samples were compared to skeletal muscles (Fig. 3). Furthermore, we visualized the distribution of the inclusion levels of the *OBSCN* exons in the studied samples (using box plots), for those exons whose at least two out of the six comparisons (Fig. 2A) produced significant results (FDR < 0.05) with $\Delta \psi>10$ (%)(Fig. 4). It is worth noting that for three of the studied exons (*i.e.* exons 48, 53 and 56) significant results were achieved in all comparisons except when postnatal hearts were compared to fetal hearts (Fig. 4, Table S1, and Fig. S3, Additional File 1).

**Fig. 5:**
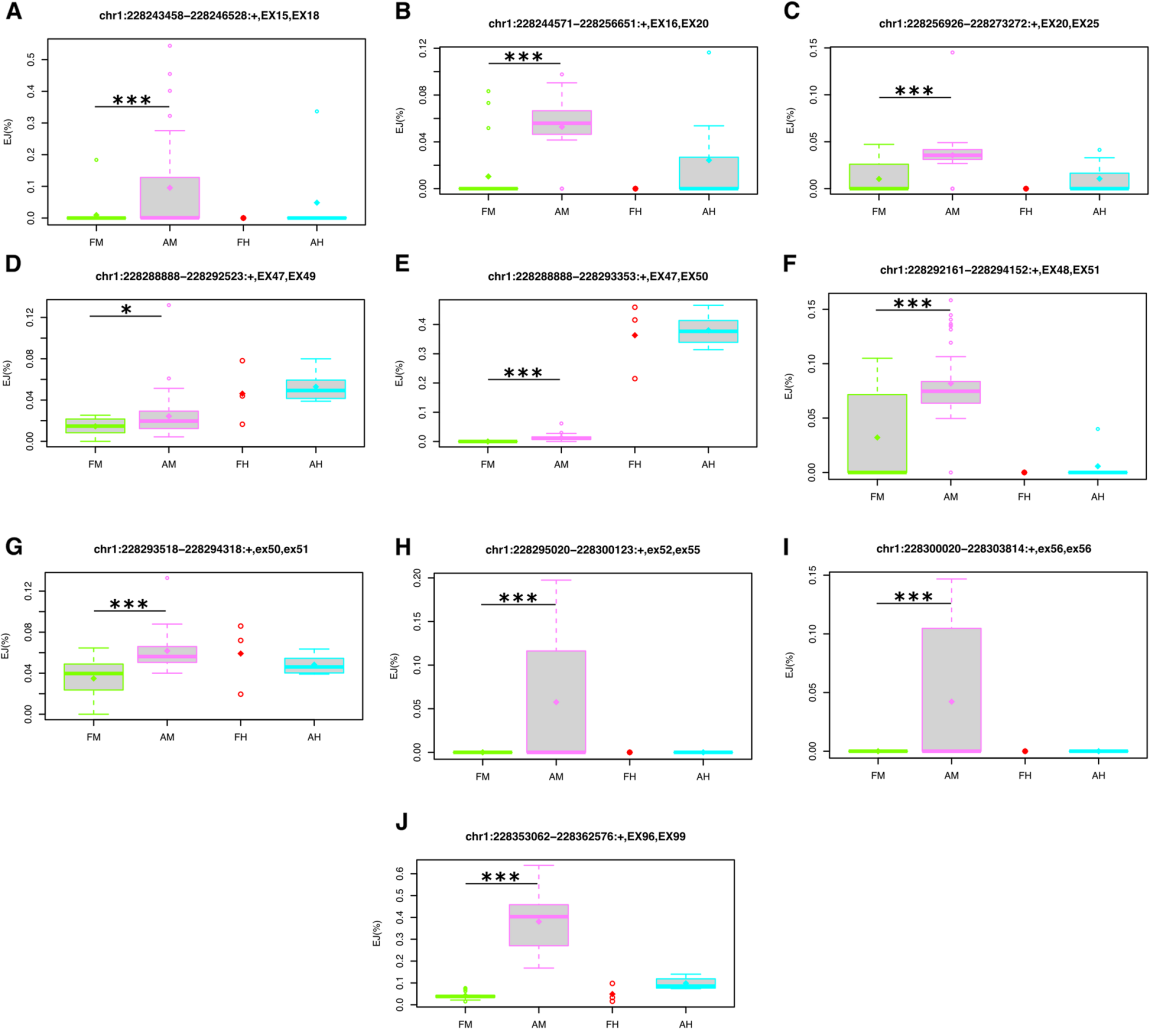
Normalized exon-exon junction levels of non-consecutive exons. Boxplots, illustrating the distribution of the normalized canonical (**A**-**G**) and non-canonical (**H**-**M**) junction levels of non-consecutive exons. If the exon-flanking 5’ or 3’ splice site is included in the reference (i.e. GENCODE) the name of the corresponding exon begins with “EX”; otherwise it starts with “ex”. The sample classes include: mostly adult postnatal muscles (AM), mostly adult postnatal hearts (AH), fetal muscles (FM), and fetal hearts (FH). The p-value and $\Delta EJ$ values for two sets of comparisons are listed below the box plots: postnatal muscle vs fetal muscle (AM > FM), and postnatal heart vs fetal heart (AH > FH). The Jonckheere Terpstra method was used to test the order and extract the significant results. The significant levels are shown using asterisks: *P* < 0.05 (*), *P* < 0.01 (**) and *P* < 0.001 (***). The box plots extend from the 25th to the 75th percentile, and the thick horizontal line represents the median. The whiskers of the boxplots show 1.5 times the interquartile range. The outliers are values higher and lower than the interquartile range

Extensive exon inclusion regulation was detected at several loci at the 5’ end (exons 17 and 18), the middle (exons 48–57) and the 3’ end of the gene (exons 97 ad 107) that were associated with skeletal muscle development (Figs. 3, 4). The inclusion (or usage) of exon 17 (*FDR(AM/FM)* = *0.00104*, $\Delta \Psi$*(AM/FM)* = *-19.2*) and exon 18 (*FDR(AM/FM)* = *0.00236*, $\Delta \Psi$*(AM/FM)* = *-18.2*) were noticeably lower in postnatal muscles compared to fetal muscles (Fig. 4A-B, 4S and Table S2). Interestingly, however, a similar effect was not seen in the postnatal heart compared to the fetal heart tissues (*FDR(AH/FH)* > *0.9*, -5% < $\Delta \Psi (AH/FH)$ < 0%). This leads us to believe that the inclusion of these exons is regulated specifically during skeletal muscle development (and not during heart development). As a result of this exon inclusion decrease, upregulation of the canonical exon junctions *chr1:228243458–228246528* (connecting exons 15 and 18) and *chr1:228244571–228256651* (connecting exons 16 and 20) were observed in postnatal muscles compared to fetal muscles (*P(AM* > *FM)* = *1e − 04, 0.0243%* ≤ $\Delta \Psi$ *EJ(AM/FM)* ≤ *0.0485%*) (Fig. 5 A-B, Table S3).

Located in the central region of the *OBSCN* gene, exons 48–56 were included significantly less in postnatal muscles than in fetal muscles (*FDR(AM/FM)* < *0.05, *$\Delta \psi$*(AM/FM)* < *-10%*) (Fig. 4). Although the inclusion levels of these exons were mostly lower in postnatal heart samples than in fetal heart samples (except for exon 52), the effects were milder and the false discovery rates were not significant (FDR(AH/FH) > 0.1, $\Delta \psi$, (AH/FH) < -10%) (Fig. 4, Table S1). We believe that the reason for observing a milder effect in heart is the small size of the fetal heart samples, as the *P*‐value for some of these effects are less than 0.05 even though their FDR values are not (Fig. 4* K-M*). Furthermore, the inclusion levels for most of these exons (*i.e.* exons 48, 49, 52–56, as well as exons 57 and 58) were significantly higher in human muscle samples compared to human heart samples (*FDR(M)* < *0.05, *$\Delta \Psi$*(M)* > *30%*), suggesting that the detection of exon inclusion variations in the cardiac muscles are technically more challenging and require more sequence reads and biological replicates (Fig. 4). Concurrent to these findings, we also noticed significant increase of several canonical as well as a few noncanonical exon junctions in postnatal muscle samples compared to fetal muscle samples (Fig. 5). The upregulated canonical exon junctions were *chr1:228288888 − 228292523* (connecting exons 47 and 49), *chr1:228288888 − 228293353* (connecting exons 47 and 50), and *chr1:228292161 − 228294152* (connecting exons 48 and 51) (Table S3)*.* The upregulated non-canonical exon junctions were *chr1:228293518 − 228294318* (overlapping exons 50 and 51), *chr1:228295020 − 228300123* (overlapping exons 52 and 55)*, and chr1:228300020 − 228303814* (overlapping exon 56) (Table S4)*.*

We developed an interactive visualization tool for the exon inclusion $\Psi$ values using the R Shiny package [11] which is available at http://psivis.it.helsinki.fi:3838/OBSCN_PSIVIS/. The software allows the users to zoom into more precise regions within the *OBSCN* gene to view the distribution of the inclusion levels (*i.e.* $\Psi )$ of the exons of interest and the measured statistics.

### Alternative first/last exons and alternative 3’ splicing

**Fig. 6:**
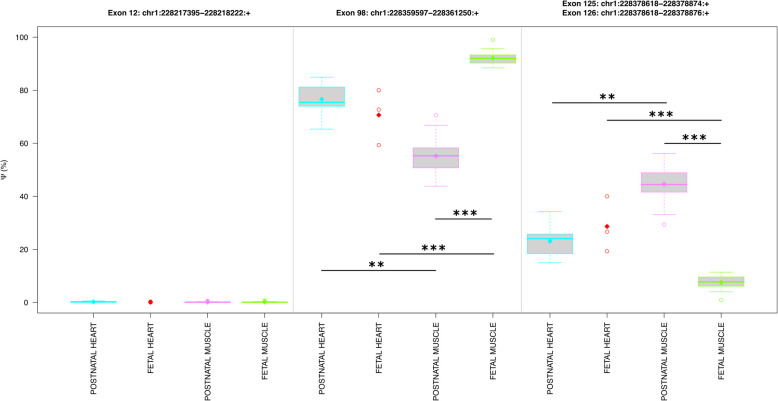
The inclusion of the alternative last exons in OBSCN mRNAs. The boxplots, illustrate the distribution of the $\Psi$ levels of the alternative last exons. The sample classes for which the $\Psi$ values are shown are: muscle vs heart (M), mostly adult postnatal vs fetal (A), postnatal muscle vs fetal muscle samples (AM/FM), postnatal heart vs fetal heart samples (AH/FH), postnatal muscle vs postnatal heart samples (AM/AH), and fetal muscle vs fetal heart samples (FM/FH). The significant levels are shown using asterisks: *P* < 0.05 (*), *P* < 0.01 (**) and *P* < 0.001 (***). They are coloured in red if $\left|\Delta \psi \right|\ge 10$. The box plots extend from the 25th to the 75th percentile, and the thick horizontal line represents the median. The whiskers of the boxplots show 1.5 times the interquartile range. The outliers are values higher and lower than the interquartile range. Since their start coordinates are identical, the $\Psi$ values for the exons 125 (i.e. 121a of meta-transcript, *chr1: 228378618–228378874*) and 126 (i.e. 121 of meta-transcript, *chr1: 228378618–228378876*) are indistinguishable, therefore reported together

We measured $\Psi$ values of the four alternative final exons (Table 2) and two alternative first exons. It is worth noting that the results for exons 126 and 125 (*i.e.* alternative last exons) were reported together as their differences are minor (Table S5). Our results showed that exon 98 (*i.e.* exon 95 of the meta-transcript) was included significantly less in the mRNAs of postnatal muscles compared to fetal muscles (*FDR(AM/FM)* = *3.574e − 19, *$\Delta \Psi$*(AM/FM)* = *− 36.74%*) (Fig. 6). In contrast, exon 125 or 126 were included significantly more (*FDR(AM/FM)* = *3.419e − 19, *$\Delta \Psi$*(AM/FM)* = *36.69%*) in the mRNAs of postnatal muscles compared to fetal muscles (Fig. 6). As a consequence, the skipping of exon 97 (together with exon 98) was significantly upregulated in the postnatal muscles compared to fetal muscles (*P(AM* > *FM)* = *1e − 04*, $\Delta$ *EJ(AM/FM)* = *0.34%*) (Figs. 5J, 4T). These findings, together with the real-time polymerase chain reaction (RT-qPCR) results, indicate a higher abundance of the longer isoform obscurin-B in the postnatal skeletal muscles despite the higher abundance of the shorter isoform obscurin-A in the fetal skeletal muscles (Fig. 4T, Table S6).

**Table 2:** The studied *OBSCN* isoforms and exons

Exon number	Meta transcript exon number	Chr	Begin	End	Strand	ENST00000284548	ENST00000422127	ENST00000493977	ENST00000570156	ENST00000660857	ENST00000680850	First exon	Last exon	Alternative splicing	Start Phase	End Phase	Domain
1	1	chr1	228208044	228208185	+				1		1	TRUE	FALSE		-	-	-
2	1a	chr1	228208063	228208185	+	1						TRUE	FALSE		-	-	-
3	1b	chr1	228208130	228208185	+		1					TRUE	FALSE		-	-	-
4	2	chr1	228211766	228212771	+	2	2		2		2	FALSE	FALSE		-	1	Ig #1, Ig #2, Ig#3
5	3	chr1	228213441	228213710	+	3	3		3		3	FALSE	FALSE		1	1	Ig #4
6	4	chr1	228214174	228214434	+	4	4		4		4	FALSE	FALSE		1	1	Ig #5
7	5	chr1	228214790	228215098	+	5	5		5		5	FALSE	FALSE		1	1	Fn3 #1
8	6	chr1	228215563	228215829	+	6	6		6		6	FALSE	FALSE		1	1	Ig #6
9	6a	chr1	228215689	228215829	+			1				FALSE	FALSE		1	1	Ig #6
10	7	chr1	228216421	228216702	+	7	7	2	7		7	FALSE	FALSE		1	1	Ig #7
11	8	chr1	228217013	228217288	+	8	8	3	8		8	FALSE	FALSE		1	1	Ig #8
12	9	chr1	228217395	228218222	+			4				FALSE	TRUE	AL=chr1:228359597-228361250:+, AL=chr1:228378618-228378874:+, AL=chr1:228378618-228378876:+	1	-	-
13	10	chr1	228219324	228219599	+				9		9	FALSE	FALSE		1	1	Ig #9
14	11	chr1	228224459	228224734	+	9	9		10		10	FALSE	FALSE		1	1	Ig #10
15	12	chr1	228243183	228243458	+	10	10		11		11	FALSE	FALSE		1	1	Ig #11
16	13	chr1	228244296	228244571	+	11	11		12		12	FALSE	FALSE		1	1	Ig #12
17	14	chr1	228245413	228245688	+	12	12		13		13	FALSE	FALSE		1	1	Ig #13
18	15	chr1	228246528	228246803	+	13	13		14		14	FALSE	FALSE		1	1	Ig #14
19	16	chr1	228249965	228250240	+	14	14		15		15	FALSE	FALSE		1	1	Ig #15
20	17	chr1	228256651	228256926	+	15	15		16		16	FALSE	FALSE		1	1	Ig #16
21	18	chr1	228259501	228259776	+				17		17	FALSE	FALSE		1	1	Ig #17
22	19	chr1	228264116	228264391	+	16	16		18		18	FALSE	FALSE		1	1	Ig #18
23	20	chr1	228268530	228268805	+	17	17		19		19	FALSE	FALSE		1	1	Ig #19
24	21	chr1	228271925	228272221	+				20		20	FALSE	FALSE		1	1	Ig #20
25	22	chr1	228273272	228273547	+				21		21	FALSE	FALSE		1	1	Ig #21
26	23	chr1	228273770	228274048	+	18	18		22		22	FALSE	FALSE		1	1	Fn3 #2
27	24	chr1	228274178	228274444	+	19	19		23		23	FALSE	FALSE		1	1	Ig #22
28	25	chr1	228274572	228274841	+	20	20		24		24	FALSE	FALSE		1	1	Ig #23
29	26	chr1	228275760	228276026	+	21	21		25		25	FALSE	FALSE		1	1	Ig #24
30	27	chr1	228276450	228276716	+	22	22		26		26	FALSE	FALSE		1	1	Ig #25
31	28	chr1	228276930	228277042	+	23	23		27		27	FALSE	FALSE		1	0	½ Ig #26
32	29	chr1	228277160	228277313	+	24	24		28		28	FALSE	FALSE		0	1	½ Ig #26
33	30	chr1	228277505	228277666	+				29		29	FALSE	FALSE		1	1	½ Ig #27
34	31	chr1	228277754	228277858	+	25	25		30		30	FALSE	FALSE		1	1	½ Ig #27
35	32	chr1	228278689	228278955	+	26	26		31		31	FALSE	FALSE		1	1	Ig #28
36	33	chr1	228279175	228279444	+	27	27		32		32	FALSE	FALSE		1	1	Ig #28 few AAs
37	34	chr1	228279821	228280087	+	28	28		33		33	FALSE	FALSE		1	1	Ig #29
38	35	chr1	228280179	228280445	+	29	29		34		34	FALSE	FALSE		1	1	Ig #30
39	36	chr1	228280530	228280796	+	30	30		35		35	FALSE	FALSE		1	1	Ig #31
40	37	chr1	228281933	228282205	+	31	31		36		36	FALSE	FALSE		1	1	Ig #32
41	38	chr1	228283018	228283284	+	32	32		37		37	FALSE	FALSE		1	1	Ig #33
42	39	chr1	228283503	228283769	+	33	33		38		38	FALSE	FALSE		1	1	Ig #34
43	40	chr1	228286078	228286350	+	34	34		39		39	FALSE	FALSE		1	1	Ig #35
44	41	chr1	228286773	228287039	+	35	35		40		40	FALSE	FALSE		1	1	Ig #36
45	42	chr1	228287694	228287960	+	36	36		41		41	FALSE	FALSE		1	1	Ig #37
46	43	chr1	228288061	228288324	+	37	37		42		42	FALSE	FALSE		1	1	Ig #38
47	44	chr1	228288625	228288888	+	38	38		43		43	FALSE	FALSE		1	1	Ig #39
48	45	chr1	228291898	228292161	+	39	39		44		44	FALSE	FALSE		1	1	Ig #40
49	46	chr1	228292523	228292786	+	40	40		45		45	FALSE	FALSE		1	1	Ig #41
50	47	chr1	228293353	228293616	+	41	41		46		46	FALSE	FALSE		1	1	Ig #42
51	48	chr1	228294152	228294415	+	42	42		47		47	FALSE	FALSE		1	1	Ig #43
52	49	chr1	228294780	228295043	+	43	43		48		48	FALSE	FALSE		1	1	Ig #44
53	50	chr1	228298454	228298717	+				49		49	FALSE	FALSE		1	1	Ig #45
54	51	chr1	228299259	228299522	+				50		50	FALSE	FALSE		1	1	Ig #46
55	52	chr1	228299882	228300145	+				51		51	FALSE	FALSE		1	1	Ig #47
56	53	chr1	228303675	228303938	+				52		52	FALSE	FALSE		1	1	Ig #48
57	54	chr1	228304303	228304566	+				53		53	FALSE	FALSE		1	1	Ig #49
58	55	chr1	228305123	228305386	+				54		54	FALSE	FALSE		1	1	Ig #50
59	56	chr1	228306372	228306638	+	44	44		55		55	FALSE	FALSE		1	1	Ig #51
60	57	chr1	228306901	228307167	+	45	45		56		56	FALSE	FALSE		1	1	Ig #52
61	58	chr1	228307259	228307531	+	46	46		57		57	FALSE	FALSE		1	1	Ig #53
62	59	chr1	228308111	228308383	+	47	47		58		58	FALSE	FALSE		1	1	Ig #54
63	60	chr1	228309099	228309295	+	48	48		59		59	FALSE	FALSE		1	0	½Ig #55
64	61	chr1	228309484	228309559	+	49	49		60	1	60	FALSE	FALSE		0	1	½Ig #55
65	62	chr1	228313614	228313886	+					2		FALSE	FALSE		1	1	Ig #56
66	63	chr1	228314814	228315080	+					3		FALSE	FALSE		1	1	Ig #57
67	64	chr1	228315847	228316119	+	50	50		61	4	61	FALSE	FALSE		1	1	Ig #58
68	65	chr1	228316709	228316984	+	51	51		62		62	FALSE	FALSE		1	1	Ig #59
69	66	chr1	228317464	228317751	+	52	52		63		63	FALSE	FALSE		1	1	Fn3
70	67	chr1	228317892	228318173	+	53	53		64		64	FALSE	FALSE		1	1	Ig #60
71	68	chr1	228318884	228319254	+	54	54		65		65	FALSE	FALSE		1	0	-
72	69	chr1	228321344	228322214	+	55	55		66		66	FALSE	FALSE		0	1	IQ, Ig #61
73	70	chr1	228323328	228323606	+	56	56		67		67	FALSE	FALSE		1	1	Ig #62
74	71	chr1	228332860	228332938	+	57	57		68		68	FALSE	FALSE		1	2	-
75	72	chr1	228333199	228333323	+	58	58		69		69	FALSE	FALSE		2	1	½ Ig #63
76	73	chr1	228333583	228333780	+	59	59		70		70	FALSE	FALSE		1	1	½ Ig #63
77	74	chr1	228334512	228334544	+	60	60		71		71	FALSE	FALSE		1	1	-
78	75	chr1	228334815	228334913	+	61	61		72		72	FALSE	FALSE		1	1	½ Ig #64
79	76	chr1	228335080	228335298	+	62	62		73		73	FALSE	FALSE		1	1	½ Ig #64
80	77	chr1	228335791	228335851	+	63	63		74		74	FALSE	FALSE		1	2	-
81	78	chr1	228336200	228336270	+	64	64		75		75	FALSE	FALSE		2	1	-
82	79	chr1	228337004	228337126	+	65	65		76		76	FALSE	FALSE		1	1	-
83	80	chr1	228337244	228337383	+	66	66		77		77	FALSE	FALSE		1	0	-
84	81	chr1	228337944	228338144	+	67	67		78		78	FALSE	FALSE		0	0	SH3
85	82	chr1	228338290	228338372	+	68	68		79		79	FALSE	FALSE		0	2	-
86	83	chr1	228338853	228339026	+	69	69		80		80	FALSE	FALSE		2	2	DH
87	84	chr1	228339945	228340101	+	70	70		81		81	FALSE	FALSE		2	0	DH
88	85	chr1	228340506	228340622	+	71	71		82		82	FALSE	FALSE		0	0	DH
89	86	chr1	228340724	228340888	+	72	72		83		83	FALSE	FALSE		0	0	DH
90	87	chr1	228341095	228341253	+	73	73		84		84	FALSE	FALSE		0	0	PH
91	88	chr1	228341437	228341614	+	74	74		85		85	FALSE	FALSE		0	1	PH
92	89	chr1	228342119	228342232	+	75	75		86		86	FALSE	FALSE		1	1	½ Ig #65
93	90	chr1	228349890	228350057	+	76	76		87		87	FALSE	FALSE		1	1	½Ig #65
94	91	chr1	228350841	228350943	+	77	77		88		88	FALSE	FALSE		1	2	1/3 Ig #66
95	92	chr1	228351321	228351450	+	78	78		89		89	FALSE	FALSE		2	0	1/3 Ig #66
96	93	chr1	228352951	228353062	+	79	79		90		90	FALSE	FALSE		0	1	1/3 Ig #66
97	94	chr1	228356151	228356193	+	80						FALSE	FALSE		1	2	-
98	95	chr1	228359597	228361250	+	81						FALSE	TRUE	AL=chr1:228217395-228218222:+, AL=chr1:228378618-228378874:+, AL=chr1:228378618-228378876:+	2	-	-
99	96	chr1	228362576	228362755	+		80		91		91	FALSE	FALSE		1	1	-
100	97	chr1	228364981	228365077	+		81		92		92	FALSE	FALSE		1	2	-
101	98	chr1	228365436	228365560	+		82		93		93	FALSE	FALSE		2	1	-
102	99	chr1	228366074	228366186	+		83		94		94	FALSE	FALSE		1	0	½ Ig #67
103	100	chr1	228366400	228366568	+		84		95		95	FALSE	FALSE		0	1	½ Ig #67
104	101	chr1	228366808	228366889	+		85		96		96	FALSE	FALSE		1	2	Kinase #1
105	102	chr1	228366975	228367175	+		86		97		97	FALSE	FALSE		2	2	Kinase #1
106	103	chr1	228367886	228367946	+		87		98		98	FALSE	FALSE		2	0	Kinase #1
107	104	chr1	228368339	228368416	+		88		99		99	FALSE	FALSE		0	0	Kinase #1
108	105	chr1	228368722	228368894	+		89		100		100	FALSE	FALSE		0	2	Kinase #1
109	106	chr1	228369136	228369162	+		90		101		101	FALSE	FALSE		2	2	Kinase #1
110	107	chr1	228369942	228370094	+		91		102		102	FALSE	FALSE		2	2	Kinase #1
111	108	chr1	228370193	228370238	+		92		103		103	FALSE	FALSE		2	0	Kinase #1
112	109	chr1	228370679	228370756	+		93		104		104	FALSE	FALSE		0	0	-
113	110	chr1	228371023	228373081	+		94		105		105	FALSE	FALSE		0	1	-
114	111	chr1	228373932	228374012	+		95		106		106	FALSE	FALSE		1	1	-
115	112	chr1	228374314	228374424	+		96		107		107	FALSE	FALSE		1	1	½ Ig #68
116	113	chr1	228374585	228374752	+		97		108		108	FALSE	FALSE		1	1	½ Ig #68
117	114	chr1	228375702	228375824	+		98		109		109	FALSE	FALSE		1	1	-
118	115	chr1	228376008	228376178	+		99		110		110	FALSE	FALSE		1	1	-
119	116	chr1	228376793	228376874	+		100		111		111	FALSE	FALSE		1	2	Kinase #2
120	117	chr1	228377052	228377291	+		101		112		112	FALSE	FALSE		2	2	Kinase #2
121	118	chr1	228377489	228377712	+		102		113		113	FALSE	FALSE		2	1	Kinase #2
122	119	chr1	228377931	228378006	+		103				114	FALSE	FALSE	A5'=chr1:228377937-228378006:+	1	2	Kinase #2
123	119a	chr1	228377937	228378006	+				114			FALSE	FALSE	A5'=chr1:228377931-228378006:+	1	2	Kinase #2
124	120	chr1	228378271	228378420	+		104		115		115	FALSE	FALSE		2	2	Kinase #2
125	121a	chr1	228378618	228378874	+		105					FALSE	TRUE	AL=chr1:228217395-228218222:+, AL=chr1:228359597-228361250:+, AL=chr1:228378618-228378876:+	2	-	Kinase #2
126	121	chr1	228378618	228378876	+				116		116	FALSE	TRUE	AL=chr1:228217395-228218222:+, AL=chr1:228359597-228361250:+, AL=chr1:228378618-228378874:+	2	-	Kinase #2

Each row of the table represents a unique OBSCN exon. The unique exon IDs based on the non-overlapping exons are listed below the “Meta-transcript exon number”. The six studied transcripts include ENST00000284548, ENST00000422127, ENST00000570156, ENST00000680850, ENST00000660857 and ENST00000493977. Below the columns labelled with the Ensembl IDs, for those exons included in the isoform, the number of the exons are stated. In the columns labelled with “First exon” and “Last exon”, the cells corresponding to the first and last exon of the isoform are marked with TRUE. The final fours columns (i.e. on the right) include the detailed information about the alternative splicing events, and the annotated domains

We also studied the alternative 3’ splicing related to the exons 122 and 123 (*i.e.* 119 and 119a of meta-transcript, respectively) (Table 2). The inclusion levels of exon 123 (i.e. 119a of meta-transcript) were very low and the upstream intron was rarely spliced across our studied samples, suggesting that almost all mRNAs in our samples included the alternative exon 122 (i.e. 119 of meta-transcript) (Fig. S4, Additional File 1﻿).

### The affected protein domains

The exons 17 and 18 that were frequently skipped in the adult skeletal muscle samples are known to code for Ig domains (Table 2). Furthermore, the exons 48–56 that were less included in the postnatal skeletal and cardiac muscles compared to the equivalent prenatal samples, also code for Ig domains (Table 2). As mentioned earlier, our results showed higher abundance of the longer *OBSCN* isoform (*e.g.* obscurin-B) compared to the shorter isoform (*e.g.* obscurin-A) in postnatal skeletal muscles, even though the shorter isoform was more abundant in fetal skeletal muscles. Compared to the short isoform (*i.e.* obscurin-A), the long isoform (*i.e.* obscurin-B) features an addition fibronectin type-III domain, two additional Ig sites and two serine/threonine type kinase sites. These variations in the domains can change the chemical/physical properties of a protein and ultimately affect its function.

### Regulation of OBSCN exon inclusion by the splicing factors

**Fig. 7:**
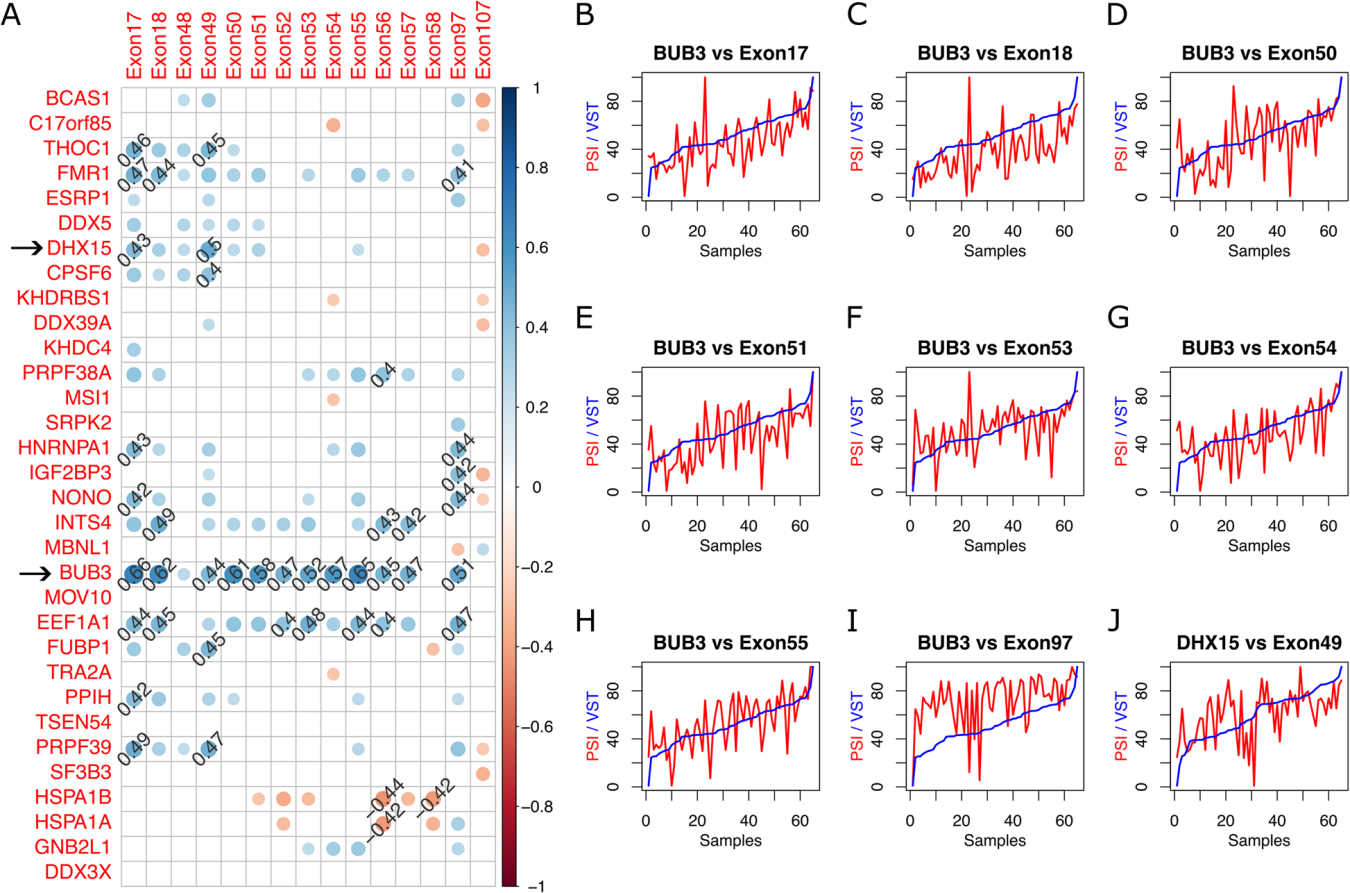
Correlation of the *OBSCN* exon inclusion levels with the expression of several splicing factors. **A** The rank correlation of the expression of the significantly differentially expressed splicing factors (comparing postnatal muscles vs fetal muscles) with the inclusion levels of the exons that were significantly differentially included (also when comparing postnatal muscles vs fetal muscles) have been shown as a matrix of circles (i.e. correlation plots). The significant (*i.e. P* < 0.05) correlations have only been shown. The size and the colour of the circles represent the correlation (*i.e. rho* value) of the corresponding splice factor (labelled on the row) with the corresponding OBSCN exon (labelled on the column). The *rho* values higher than 0.4 or lower than -0.4 have also been written. **B**-**J** For a highly correlated pair (*i.e. |rho|*> 0.5, *P* < 0.05), the two lines in the plot show the PSI values of the OBSCN exon, as well as the VST normalized expression levels of the splicing factor (scaled to 100) across the studied samples

We examined the Spearman (rank) correlation of the expression of the significantly differentially expressed splicing factors (when comparing postnatal to fetal muscle samples) with the inclusion $\Psi$ values of the *OBSCN* exons that were significantly differentially included (in postnatal muscle vs fetal muscle) across the studied skeletal muscle samples. Several significant correlations (|*rho*|> 0.4, *P* < 0.05) were detected, *e.g.* expression of *DHX15*, *THOC1*, PRPF1 with inclusion levels of exon 17 and 49 (Fig. 7A-J, Table S7-S10). However, remarkably the expression of *BUB3* was significantly correlated with the inclusion levels of most of the significantly differentially included exons (Fig. 7A-I). The BUB3 gene belongs to the budding uninhibited by benomyl (BUB) protein family and is involved in mitosis, aging, carcinogenesis, as well as splicing [12, 13]. Our results suggest the possibility of regulation of *OBSCN* splicing by *BUB3* especially during muscle development.

## Discussion

Alternative splicing plays an essential role in the regulation of gene expression during organ development in mammalians. It is known that throughout the different stages of human life, a great number of genes are differentially spliced, especially in tissues such as brain and heart [14]. Here we studied *OBSCN*, a gene associated with neuromuscular function that has 121 non-overlapping (or 126 unique) exons and codes for some of the largest mRNAs in the human genome. *OBSCN* is upregulated in aged skeletal muscle myofiber fragments (*e.g.* MF-IIsc) and RASA4 + myocytes (Table S10) [15]. However, we studied the splicing regulation of *OBSCN* during human skeletal and cardiac muscle development. Given the large number of exons in the gene, we hypothesized that it undergoes extensive alternative splicing regulation during muscle and heart development. As a result, we discovered several alternative splicing events in *OBSCN* associated with skeletal and cardiac muscle development. These mainly included cassette exons and alternative last exon usage events that were significantly differential in the postnatal human skeletal and cardiac muscles, compared to the equivalent prenatal tissues.

The splicing event that was most frequently differential across our pre- and postnatal cardiac, and skeletal muscle samples was exon inclusion (Figs. 3 and 4). The predominance of the exon inclusion (and exon skipping) was not surprising as in previous studies this splicing event has been the most frequent out of all the significant alternative splicing events found in mammals and vertebrates [16]. Also in-line with these findings, exon skipping has been reported as the most frequently regulated event during the development of seven organs (including heart) in six mammals (including human) and a bird [14]. In mice muscles, extensive differential gene expression and alternative splicing has been discovered to occur during the first two weeks after birth, with the vast majority of these alternative splicing events (*i.e.* 77%) being exon skipping [17].

In this study, we discovered extensive exon inclusion regulation at several loci, at the 5’ end, the middle and the 3’ end of *OBSCN* gene that are associated with cardiac or skeletal muscle development. Exons 48–56 of *OBSCN* were significantly less included in RNAs in the postnatal muscles compared to the fetal muscles (*FDR(AM/FM)* < *0.05, *$\Delta \Psi$*(AM/FM)* < *-10%*) (Fig. 4C-M). A similar, albeit milder, effect was also seen in cardiac muscles (*FDR(AH/FH)* > *0.05, *$\Delta \Psi$*(AH/FH)* < *0%*) (Fig. 4C-M). It is worth mentioning that from this region of *OBSCN* (*i.e.* exons 48–56), exons 48–54 have previously been reported to undergo developmentally dynamic alternative splicing, especially during human heart development [14]. A dynamic alternative splicing event is a splicing event whose PSI changes in the studied tissue (e.g. heart) during human life is greater than 20% [14]. Another exon inclusion regulation, exhibiting strong effects in human muscle development and mild effects in human heart development, was seen *in* exons 17 and 18 (Fig. 4A,B). Additionally we noticed significant increase in several canonical as well as a few non-canonical exon junction levels in postnatal muscles compared to fetal muscles. To our knowledge the association of these alternative splicing events with human skeletal muscle development has not previously been reported. We also discovered several splicing factors (*e.g. DHX15*, *THOC1*, *PRPF1, BUB3)* whose expression levels were significantly correlated (*P* < *0.05* and *|rho|*≥ *0.4*) with the inclusion levels of the significantly differentially included exons (when comparing postnatal to fetal muscles) (Fig. 7A-J). Our results suggest that the differential inclusion of the *OBSCN* exons during skeletal muscle development may be regulated by *BUB3*. In fact, Bub3 and BuGZ are two essential mitotic regulators that together interact with the splicing machinery in the interphase nucleus [13]. Silencing of either Bub3 or BuGZ has previously shown to enhance exon skipping in Human foreskin fibroblast (*i.e.* HFF) and ovarian carcinoma TOV21G cell lines [13]. Furthermore, BugZ was not differentially expressed (in postnatal skeletal muscles compared to prenatal skeletal muscles), therefore it is likely that the exon inclusion effects that we report are caused by the differential expression of Bub3 (Table S10). However, it is worth noting that due to scarcity of prior knock-down studies, especially in human muscle samples, a thorough analysis (beyond the scope of this study) is necessary before the precise role of Bub3 in RNA splicing, in muscles of human and other species can be concluded.

In addition to exon skipping, we discovered an alternative last exon usage event that is associated with skeletal muscle development. We discovered that the *OBSCN* isoform that ends with exon 98 (e.g. obscurin-A) is expressed much higher in fetal skeletal muscles, whereas the larger isoform that skip exons 97 and 98 and end with either exon 125 or exon 126 (e.g. obscurin-B) is more expressed in postnatal skeletal muscles (Fig. 4T and 6). To our knowledge the higher abundance of the longer *OBSCN* isoform in postnatal muscles compared to fetal muscles has neither been reported earlier.

Almost all the significantly differentially included exons detected in our study code for immunoglobulin domains. Similar to that in titin, obscurin feature long repeats of Ig domains (Table 2) and these tandem Ig domains are mostly coded by individual exons (Table 2) [6, 18]. Therefore, not surprisingly, the most affected (*i.e.* skipped) exons in our samples code for a complete (not partial) Ig domain (Table 2, Fig. 3). Even though the function of the repeated Ig domains in sarcomeric genes (*e.g. OBSCN* and *TTN*) has not thoroughly been studied previously, the extended tandem Ig domains are known to associate with increased elasticity in the isoforms [19]. Furthermore, this has been described as the reason that the sarcomere in skeletal muscle is more elastic comparted to the sarcomere in cardiac muscle [20].

The N-terminus of obscurin interacts with several proteins such as titin, slow myosin binding protein C, and myomesin. Furthermore, the 58th and 59th Ig domains in obscurin (coded by exons 67 and 68 of *OBSCN*, with ~ 100% of exon inclusion rate) are known to interact with the Z-band of titin, signifying the essential role of *OBSCN* in myofibrillogenesis [1, 21]. Further structural studies are needed to reveal the precise effects of the significantly differential exon inclusion events that we have described above. However, as the exons 48–58 are distanced from the 5’ end and the titin interacting sites (*i.e.* exons 67 and 68), the skipping of these exons neither is expected, nor has previously been reported to directly affect the interaction of the obscurin N-terminal with other proteins or to affect the obscurin-titin interaction. The C-terminus of obscurin-A interacts with small Ankyrin 1 (sAnk1) and Ankyrin-B [22, 23]. These interactions are essential to the Ca2 + homeostasis and the assembly of the dystrophin complex, respectively [21]. Furthermore, changes in calcium homeostasis and reduction of dystrophin have both been reported in aged skeletal muscles [24, 25]. However, even though it can be speculated that the downregulation of obscurin-A in adult skeletal muscles (compared to fetal skeletal muscles) that we have described above may contribute to these phenotypes, the connection of these phenotypes to obscurin splicing has not specifically been studied.

Finally, RNA splicing regulation information can assist the researchers and the clinicians to understand the clinical impacts of the exonic variants. As for instance we have recently shown how similar exon usage information for *TTN* can be used to explain the disease course in nine titinopathy patients [26]. Remarkably, the exon usage information has also been useful in ruling out titinopathy diagnosis for a prenatal case [26]. Therefore, we believe that information related to the *OBSCN* exon usage and splicing regulation during skeletal/cardiac muscle development, that we have described in detail here, is potentially useful for clinical interpretation of the exonic mutations in *OBSCN*.

## Conclusion

In this study we have described several novel exon skipping events that are associated with human cardiac and skeletal muscle development. Additionally, we discovered an alternative final exon usage event associated with human skeletal muscle development. This information allows us to understand the regulation of *OBSCN* splicing during human muscle and heart development. Furthermore, the data is essential for clinical and prognostic interpretation of the *OBSCN* exonic variants and understanding the effects of these variants on the protein expression in different stages of life*.*

Our study is strengthened by the thoroughness of the analysis and the support for P-values (and FDRs) that describe how significantly differential the alternative splicing events are during human skeletal and cardiac muscle development. The study is however reliant on the analysis of RNAseq data from a limited selection of muscle types (*e.g.* tibialis anterior and vastus lateralis from the studied postnatal individuals). Therefore, it can be improved by including RNAseq data from more samples and from a more diverse types of muscle tissues in the analysis. Finally, we have developed an interactive visualization tool (using the shiny R package) that can easily be used by the clinicians to check the inclusion level of each *OBSCN* exon during skeletal and cardiac muscle development. The interactive R shiny application is available at http://psivis.it.helsinki.fi:3838/OBSCN_PSIVIS/.

## Methods

### In-house data

For prenatal analysis, a trained fetal pathologist collected fetal skeletal muscles (*n* = 20) and fetal cardiac muscles (*n* = 2) from 2 different fetuses, without muscle pathology, obtained from voluntary termination of pregnancy (TOP).

For postnatal analysis, we collected sample biopsies from an internal cohort of 44 individuals (Table 1). RNA was extracted with the Qiagen RNeasy Plus Universal Mini Kit (Qiagen, Hilden, Germany) according to the instructions provided by the manufacturer. Total RNA-Seq libraries were prepared using the Illumina Ribo-Zero Plus rRNA Depletion Kit (Illumina, Palo Alto, CA, USA) at the Oxford Genomics Center, Welcome Trust Institute, Oxford, United Kingdom and Novogene. Sequencing was performed using NovaSeq 6000 (Illumina), generating over 80 million 150 bp-long reads per sample.

### External data

In addition to our in-house data, we studied four samples from ENCODE with accession IDs: ENCBS067RNA (fetal skeletal muscle tissue, 19 weeks female), ENCBS068RNA (fetal skeletal muscle tissues, 22 weeks male), ENCBS055RNA (fetal heart tissue, 28 weeks female) and ENCBS056RNA (fetal heart tissue, 19 weeks female) [27, 28]. Furthermore, for a view on *OBSCN* expression across different human tissues, we obtained data from the GTEx Portal (Analysis Release V8) and dbGaP accession number phs000424.v8.p2 on 09/13/23.

### RNA-Seq read alignment

The paired RNA-Seq reads were mapped to the Human Genome (GRCh38.p13) using the splice-aware alignment software STAR (V2.7.7a) [29]. The software was run in 2-pass mode and most parameters were set to their default values. For the gene annotation, Gencode.v39 was used (further details available in supplemental methods, Additional file 1).

### Splicing analysis and exon inclusion level estimation

The inclusion levels (i.e. PSI or Ψ values) of all unique exons in human genome, including those of OBSCN gene, were measured using the Intron Exon Retention Estimator (IntEREst) R/Bioconductor package (V1.26.1) [30]. IntEREst is a comprehensive RNA-Seq read summarization, differential intron retention and splicing analysis software. It supports tools that measure suitable Ψ values and run statistical differential test for splicing analysis. The inclusion Ψ values were measured for every OBSCN exon. The statistical significance of the increase or decrease of the inclusion levels of OBSCN exons was preformed genome-wide for all exons (except for the first and last exons), however later the results for the OBSCN exons were extracted [31]. The statistical test compared the variation of inclusion of each exon relative to the genome-wide variation observed for inclusion of the studied exons. The analysis was adjusted for possible biases introduced by the different sequencing batches by including this parameter as a covariate in the design model of the statistical tests (further details available in supplemental methods, Additional file 1).

In addition to exon skipping/inclusion, we ran a similar analysis for the inclusion of the alternative first and last exons, as well as the only case of alternative 3’ splicing in *OBSCN* (*i.e.* affecting exons 122 and 123, or 119 and 119a from the meta transcript) (Table 2). All the first/last exons of the studied genes (including *OBSCN*) were extracted using biomaRt [32]. All *P*-values were adjusted for multiple testing, using the Benjamini–Hochberg method [33]. An FDR < 0.05 cutoff was used to extract the significant results.

Finally, differential gene expression analysis was performed using DESeq2. The analysis was adjusted for the batch effects. The rank correlation of the VST normalized expression levels of the splicing factors (whose IDs were extracted from other studies [34–36]) with the exon inclusion $\Psi$ values was also measured using the Spearman method (further details available in supplemental methods, Additional file 1).

### Real-time polymerase chain reaction (RT-qPCR) validation

RNA was extracted from two adult muscle samples and two fetal muscle samples using the Qiagen RNeasy Plus Universal Mini Kit (Qiagen, Hilden, Germany) and according to the instructions provided by the manufacturer. The cDNA synthesis was performed using SuperScript III Reverse Transcriptase (Invitrogen TM) and random primers, according to the protocol provided by the manufacturer. The UCSC In-Silico PCR tool and Primer3web v4.1.0 were used to design primers to target either exon-exon junctions or other regions near the junctions (Table S11). The RT-qPCR assays were performed using the iQ SYBR Green Supermix (BIO-RAD) and 25 nM of each specific primer. Furthermore, three technical replicates were taken into consideration. For the normalization, 18S was used as the reference gene. The final results were calculated using the ΔΔCt method and the relative quantification values were plotted (Fig. 4S-T).

## Supplementary Information

Additional file 1: Supplemental methods and Figures S1-4.

Additional file 2: Table S1. Inclusion levels of OBSCN exons.

Additional file 3: Table S2. RT-qPCR of inclusion level changes of OBSCN exons in fetal and post-natal skeletal muscle samples.

Additional file 4: Table S3. Canonical exon-exon junction level changes (for non-consecutive exons).

Additional file 5: Table S4. Non-canonical exon-exon junction level changes (for non-consecutive exons).

Additional file 6: Table S5. Alternative last exon PSI value changes.

Additional file 7: Table S6. RT-qPCR of inclusion level changes of long vs short OBSCN isoforms in fetal and post-natal skeletal muscle samples.

Additional file 8: Table S7. The studied splicing factors.

Additional file 9: Table S8. Spearman (rank) correlation of expression of splicing factors with exon inclusion levels PSI values across the skeletal muscle samples.

Additional file 10: Table S9. Spearman (rank) correlation of expression of splicing factors with exon inclusion levels PSI values across the cardiac muscle samples.

Additional file 11: Table S10. Differential expression analysis results for OBSCN and the studied Splicing Factors.

Additional file 12: Table S11. Primer information for the qPCR validation.

## Data Availability

The in-house RNA-Seq data analysed during the current study are available in the Gene Ex-pression Omnibus repository under accession number GSE270408 (https://www.ncbi.nlm.nih.gov/geo/query/acCite Reference.c.cgi?acc=GSE270408). Due to GDPR the intron and exon level read count tables are publicly available, however further data are available from the corresponding author upon reasonable request. The external RNA-Seq data analysed during the current study are available in the ENCODE repository under accession numbers ENCFF001RMW, ENCFF001RMX, ENCFF001RPA, ENCFF001RPB, ENCFF001ROH, ENCFF001ROG, ENCFF001RPf and ENCFF001RPE (accessible via https://www.encodeproject.org/experiments/ENCSR000AEZ/ and https://www.encodeproject.org/experiments/ENCSR000AFF/) [27, 28]. The analysis scripts are available in GitHub (accessible via https://github.com/gacatag/OBSCN_SCRIPTS).
