# Supplementary material for: OBSCN undergoes extensive alternative splicing during human cardiac and skeletal muscle development
Source: Skelet Muscle. 2025 Mar 1;15:5. doi: 10.1186/s13395-025-00374-6 (PMC11871629; doi:10.1186/s13395-025-00374-6)
Supplement: Supplementary file 1 — Additional file 1: Supplemental methods and Figures S1-4. [file 13395_2025_374_MOESM1_ESM.pdf]

OBSCN undergoes extensive alternative splicing during human cardiac and skeletal muscle development

Ali Oghabian[1,2], Per Harald Jonson[1,3], Swethaa Natraj Gayathri[1,3], Mridul Johari[1,4], Ella Nippala[1], David Gomez Andres[5], Francina Munell[5], Jessica Camacho Soriano[6], Maria Angeles Sanchez Duran[7], Juha Sinisalo[8], Heli Tolppanen[9], Johanna Tolva[10], Peter Hackman[1,3], Marco Savarese[1,3]*, Bjarne Udd[1,3,5, 11]*

1 Folkhälsan Research Center, Helsinki, Finland

2 Research Program for Clinical and Molecular Metabolism, Faculty of Medicine, University of Helsinki, 00014 Helsinki, Finland.

3 Department of Medical Genetics, Medicum, University of Helsinki, Helsinki, Finland

4 Harry Perkins Institute of Medical Research, Centre for Medical Research, University of Western Australia, Nedlands, WA, Australia.

5 Pediatric Neuromuscular Unit. Child Neurology Department. Hospital Universitari Vall d'Hebron, Vall d'Hebron Research Institute (VHIR) ES.

6 Histology Department, Vall d'Hebron University Hospital ES.

7 Prenatal Diagnosis Department, Vall D'hebron University Hospital ES.

8 Maternal Fetal Medicine Unit, Department of Obstetrics, Universitat Autònoma de Barcelona, Hospital Vall D'hebron, Barcelona, Spain

9 Helsinki University Central Hospital, Helsinki, Finland

10 Transplantation Laboratory, Department of Pathology, University of Helsinki, Finland

11 Neuromuscular Research Center, Department of Neurology, Tampere University and University Hospital, Tampere, Finland

*Equal contribution **Corresponding author:** Ali Oghabian (ali.oghabian@helsinki.fi)

Additional File 1: Supplemental methods and Figures S1-4

Supplemental methods

Figure S1. The distinction of samples of different sexes and clinical diagnoses based on *OB-SCN* exon inclusion.

Figure S2. Heatmap plot of inclusion levels of the studied exons of *OBSCN*.

Figure S3. Inclusion levels of the studied exons of *OBSCN*.

Figure S4. Alternative 3’ splicing.

Supplemental methods

In-house data

For prenatal analysis, a trained fetal pathologist collected fetal skeletal muscles (n=20) and fetal cardiac muscles (n=2) from 2 different fetuses, without muscle pathology, obtained from voluntary termination of pregnancy (TOP).

For postnatal analysis, we collected sample biopsies from an internal cohort of 44 individuals (Table 1).

RNA was extracted with the Qiagen RNeasy Plus Universal Mini Kit (Qiagen, Hilden, Germany) according to the instructions provided by the manufacturer. Total RNA-Seq libraries were prepared using the Illumina Ribo-Zero Plus rRNA Depletion Kit (Illumina, Palo Alto, CA, USA) at the Oxford Genomics Center, Welcome Trust Institute, Oxford, United Kingdom and Novogene. Sequencing was performed using NovaSeq 6000 (Illumina), generating over 80 million 150bp-long reads per sample.

External data

In addition to our in-house data, we studied four samples from ENCODE with accession IDs: ENCBS067RNA (fetal skeletal muscle tissue, 19 weeks female), ENCBS068RNA (fetal skeletal muscle tissues, 22 weeks male), ENCBS055RNA (fetal heart tissue, 28 weeks female) and ENCBS056RNA (fetal heart tissue, 19 weeks female) [27,28]. Furthermore, for a view on *OBSCN* expression across different human tissues, we obtained data from the GTEx Portal (Analysis Release V8) and dbGaP accession number phs000424.v8.p2 on 09/13/23.

RNA-Seq read alignment

The paired RNA-Seq reads were mapped to the Human Genome (GRCh38.p13) using the splice-aware alignment software STAR (V2.7.7a) [29]. All parameters relevant to the alignment process were set to their default settings except for *--outSAMattributes* that was set to *ALL, --sjdbScore* to *1, --limitOutSJcollapsed* to *2000000*, and *--outMultimapperOrder* to

Random. Furthermore, for a more sensitive exon junction processing (which leads to increased read counts for the exon junctions), the software was run in 2-pass mode by setting the *--twopassMode* parameter to *Basic*. For the gene annotation, Gencode.v39 was used.

Splicing analysis and exon inclusion level estimation

The inclusion levels (*i.e.* PSI or Ψ values) of all unique exons in human genome, including those of *OBSCN* gene, were measured using the Intron Exon Retention Estimator (IntEREst) R/Bioconductor package (V1.26.1) [30]. IntEREst is a comprehensive RNA-Seq read summarization, differential intron retention and splicing analysis software. It supports methods to summarize the reads that span the introns, map to the introns/exons, skip the exons, *etc*. It also supports tools that measure suitable Ψ values and run statistical differential test for splicing analysis. The inclusion Ψ values were measured for every *OBSCN* exon. It is 100 times the fraction of the reads that span the flanking introns (*i.e.* IS), to the sum of this number with twice the number of reads that skip the exon (*i.e.* ES) (*formula 1*). The measured Ψ (in percentage scale) is a value in the range of [0,100]. If all reads support the inclusion of an exon (i.e. splicing of the spanning introns) then Ψ would equal to 100. On the contrary, if all reads skip the exon, Ψ would be zero. Note that we exclusively considered the paired reads, which both pairs were mapped within the genome coordinates of the *OBSCN* gene. This was to exclude any reads from the gene fusions.

$$\Psi_{IR} = 100 \times \frac{IS}{IS + 2 \times ES} \quad (\textit{formula 1})$$

The statistical significance of the increase or decrease of the inclusion levels of *OBSCN* exons (for all exons except for the first and last exons of the studied transcripts) was estimated using the *deseqInterest()* function [30], *i.e.* a *DESeq2* dependent differential splicing analysis function supported by IntEREst that assumes that the read counts follow a negative binomial distribution [31]. The differential exon inclusion analysis was performed genome-wide for all exons, however later the results for the *OBSCN* exons were extracted. The statistical test

compared variations in the inclusion of each exon relative to the genome-wide variation observed for the inclusion of the exons. The analysis was adjusted for possible biases introduced by the different sequencing batches by including this parameter as a covariate in the design model of the statistical tests. For PCA analysis, the *prcomp()* function of the *stats* package of R (V3.4.0) was used. For the heatmap plots, the *heatmap.2()* function supported by the *gplots* package (V3.1.3.1) was used, whilst the Euclidean distance was used for hierarchical clustering.

In addition to exon skipping/inclusion, we analyzed the inclusion levels of the alternative first and last exons. All exons that were annotated as the first/last exon of the studied isoforms were extracted using biomaRt [32]. For genes other than *OBSCN*, the analysis was restricted to isoforms with RefSeq ID annotations. This was to avoid analyzing many isoforms with incomplete annotated 5' or 3' ends. It is worth noting that the Ψ values of these exons cannot be estimated with the same method that was used for the other exons (*formula 1*) as they lack exon-skipping sequence reads. Instead, for these exons, we define Ψ values specific to the inclusion of alternative first (AF) and last (AL) exons *(formula 2)*. These values can be measured for any first (and last) exon of an isoform for which at least one alternative first (and respectively last) exon is featured in other isoforms. For any gene with *M* known first exons, Ψ_{AF_n} is the number of reads that span the downstream intron of the *n*th exon relative to the sum of reads that span the downstream introns of all alternative first exons, in percentile scale *(formula 2)*. A similar benchmark was used for the alternative last exons, with the exception that the reads spanning the upstream introns were taken into account to measure Ψ *(formula 2)*. Furthermore, similar to the exon inclusion analysis, *DESEq2* was used to analyze the statistical significance of the Ψ changes genome-wide. We compared these values in the postnatal heart and postnatal muscle samples to fetal heart and fetal muscle samples, respectively.

All P-values were adjusted for multiple testing using the Benjamini-Hochberg method [33]. An FDR < 0.05 cutoff was used to extract the significant results.

$$\Psi_{AF_n} = 100 \times \frac{IS_n}{\sum_{m=1}^{M} IS_m} \text{ (formula 2)}$$

Finally, for the only two exons annotated with alternative 3' splicing of upstream intron, *i.e.* exons 122 and 123 (or 119 and 119a from the meta transcript) (*Table 2*), we used a method similar to that we used for alternative last exons (formula 2) that measures the Ψ values based on the reads that span the upstream introns. Differential gene expression analysis was performed using DESeq2. We also adjusted for the possible batch effects. The rank correlation of VST normalized expression of the splicing factors (whose IDs were extracted from other studies [34–36]) with the Ψ values was also measured using the Spearman method. The gene expression levels were also adjusted for batch effects using the ComBat function of the 'sva' R/Biocoducntor package.

Real-time polymerase chain reaction (RT-qPCR) validation

RNA was extracted from two adult muscle samples and two fetal muscle samples using the Qiagen RNeasy Plus Universal Mini Kit (Qiagen, Hilden, Germany) and according to the instructions provided by the manufacturer. The cDNA synthesis was performed using SuperScript III Reverse Transcriptase (Invitrogen TM) and random primers, according to the protocol provided by the manufacturer. The UCSC In-Silico PCR tool and Primer3web v4.1.0 were used to design primers to target either exon-exon junctions or regions near the junctions. The RT-qPCR assays were performed using the iQ SYBR Green Supermix (BIO-RAD) and 25nM of each specific primer. Furthermore, three technical replicates were taken into consideration. For the normalization, 18S was used as the reference gene. The final results were calculated using the ΔΔCt method and the relative quantification (RQ) values were plotted (*Fig. 4S-T*).

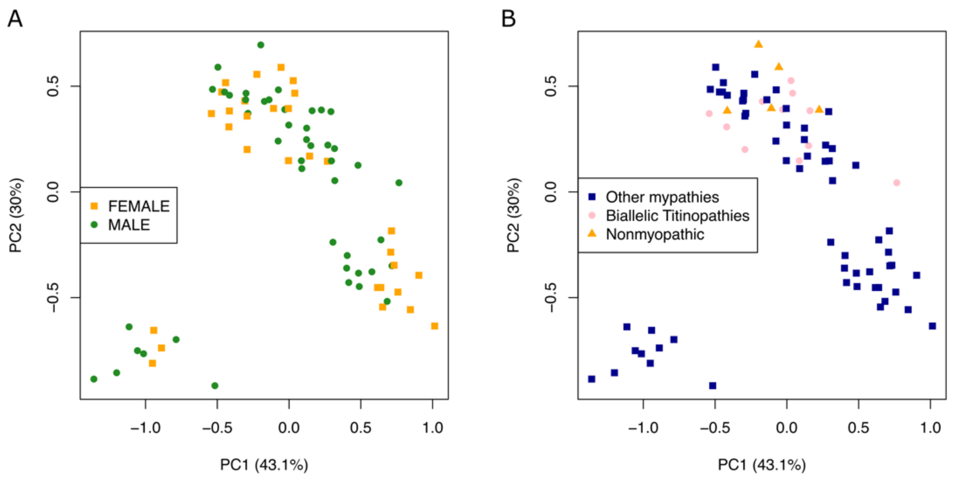

Figure S1. The distinction of samples of different sexes and clinical diagnoses based on *OBSCN* exon inclusion: The distinction of the studied sample groups based on the PC1 and PC2 dimensions. These dimensions were extracted from PCA analysis of the Percentage Spliced In (PSI) inclusion levels of exons of OBSCN across the studied samples. Each dot represents a sample and its colour and shape represent its corresponding group. The percentage of the variance within the data that is explained by the PC dimension is stated in parentheses in the axis labels. The dots have been coloured and distinguished based on 1) sex and 2) clinical diagnosis of the studied individuals.

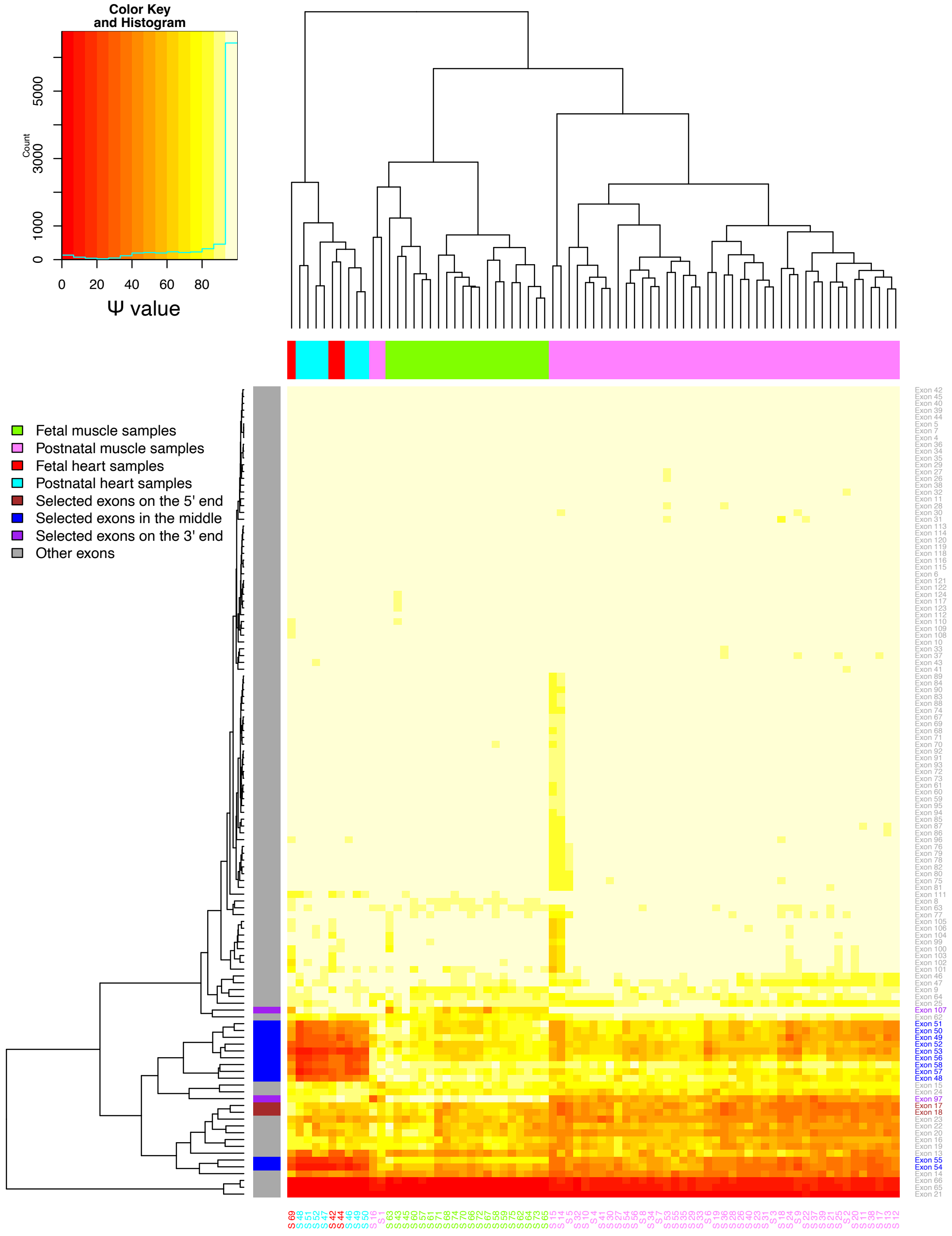

Figure S2. Heatmap plot of inclusion levels of the studied exons of *OBSCN*: The Percentage Spliced In (*i.e.* PSI or *Ψ*) inclusion level of the studied *OBSCN* exons in the studied samples is shown in the heatmap. The inclusion levels of the first and last exons of the transcripts are not shown as their measurements are not accurate. The colour key for the heatmap is shown in a box at the top left of the figure. The light-blue coloured line that goes through the

histogram shows the frequency of the Ψ values. The sample and the exon groups are labelled with distinctive colours. The exons are coloured based on how they were selected in the downstream analysis. The exons were selected if they were detected as significantly differentially spliced (FDR < 0.05) in at least three of the comparisons performed in the study. The Euclidean distance was used for hierarchical clustering.

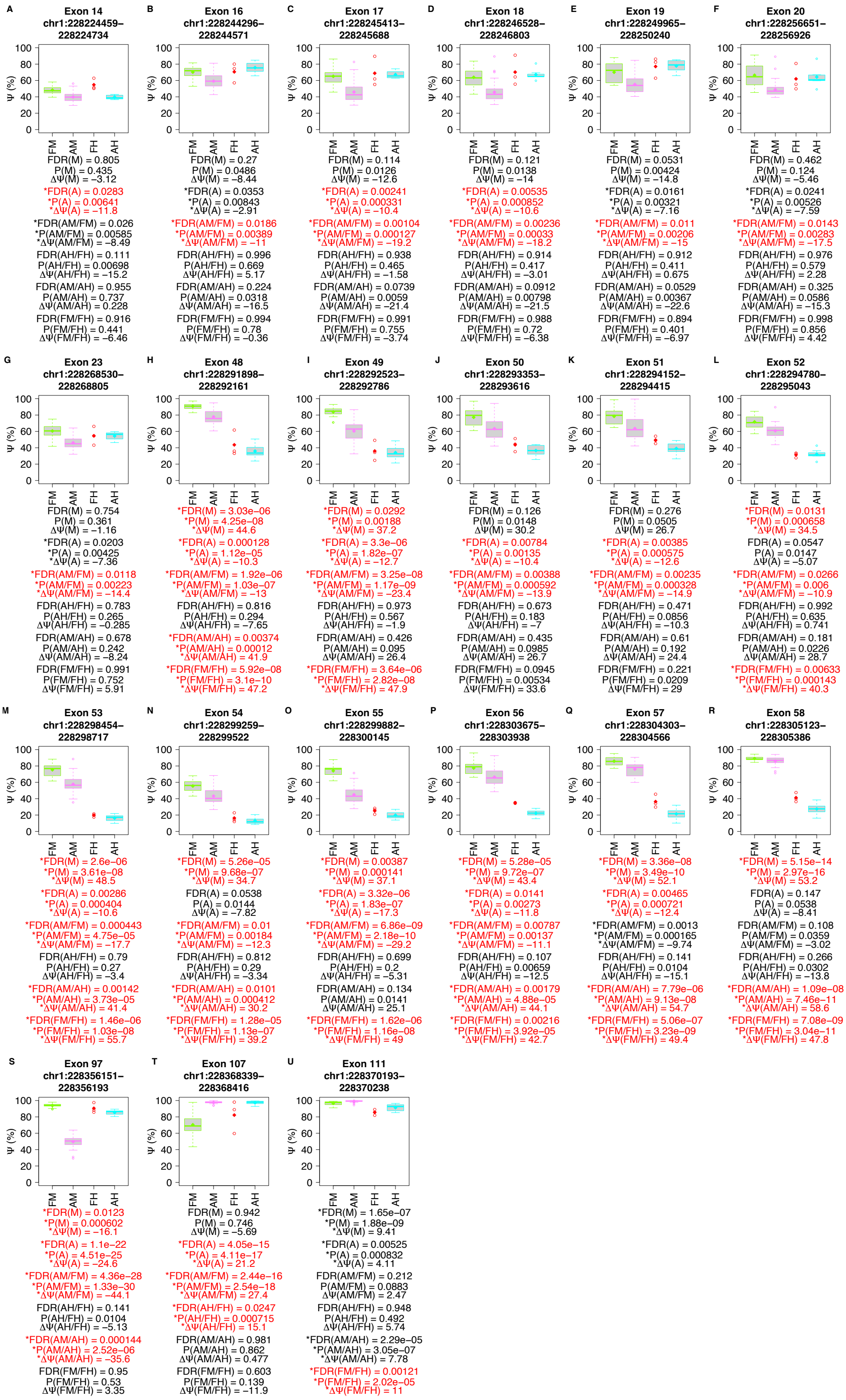
A
Exon 14
chr1:228224459–228224734
Ψ (%)
FM AM FH AH
FDR(M) = 0.805
P(M) = 0.435
ΔΨ(M) = −3.12
*FDR(A) = 0.0283
*P(A) = 0.00641
*ΔΨ(A) = −11.8
*FDR(AM/FM) = 0.026
*P(AM/FM) = 0.00585
*ΔΨ(AM/FM) = −8.49
FDR(AH/FH) = 0.111
P(AH/FH) = 0.00698
ΔΨ(AH/FH) = −15.2
FDR(AM/AH) = 0.955
P(AM/AH) = 0.737
ΔΨ(AM/AH) = 0.228
FDR(FM/FH) = 0.916
P(FM/FH) = 0.441
ΔΨ(FM/FH) = −6.46
B
Exon 16
chr1:228244296–228244571
FDR(M) = 0.27
P(M) = 0.0486
ΔΨ(M) = −8.44
*FDR(A) = 0.0353
*P(A) = 0.00843
*ΔΨ(A) = −2.91
*FDR(AM/FM) = 0.0186
*P(AM/FM) = 0.00389
*ΔΨ(AM/FM) = −11
FDR(AH/FH) = 0.996
P(AH/FH) = 0.669
ΔΨ(AH/FH) = 5.17
FDR(AM/AH) = 0.224
P(AM/AH) = 0.0318
ΔΨ(AM/AH) = −16.5
FDR(FM/FH) = 0.994
P(FM/FH) = 0.78
ΔΨ(FM/FH) = −0.36
C
Exon 17
chr1:228245413–228245688
FDR(M) = 0.114
P(M) = 0.0126
ΔΨ(M) = −12.6
*FDR(A) = 0.00241
*P(A) = 0.000331
*ΔΨ(A) = −10.4
*FDR(AM/FM) = 0.00104
*P(AM/FM) = 0.000127
*ΔΨ(AM/FM) = −19.2
FDR(AH/FH) = 0.938
P(AH/FH) = 0.465
ΔΨ(AH/FH) = −1.58
FDR(AM/AH) = 0.0739
P(AM/AH) = 0.0059
ΔΨ(AM/AH) = −21.4
FDR(FM/FH) = 0.991
P(FM/FH) = 0.755
ΔΨ(FM/FH) = −3.74
D
Exon 18
chr1:228246528–228246803
FDR(M) = 0.121
P(M) = 0.0138
ΔΨ(M) = −14
*FDR(A) = 0.00535
*P(A) = 0.000852
*ΔΨ(A) = −10.6
*FDR(AM/FM) = 0.00236
*P(AM/FM) = 0.00033
*ΔΨ(AM/FM) = −18.2
FDR(AH/FH) = 0.914
P(AH/FH) = 0.417
ΔΨ(AH/FH) = −3.01
FDR(AM/AH) = 0.0912
P(AM/AH) = 0.00798
ΔΨ(AM/AH) = −21.5
FDR(FM/FH) = 0.988
P(FM/FH) = 0.72
ΔΨ(FM/FH) = −6.38
E
Exon 19
chr1:228249965–228250240
FDR(M) = 0.0531
P(M) = 0.00424
ΔΨ(M) = −14.8
*FDR(A) = 0.0161
*P(A) = 0.00321
*ΔΨ(A) = −7.16
*FDR(AM/FM) = 0.011
*P(AM/FM) = 0.00206
*ΔΨ(AM/FM) = −15
FDR(AH/FH) = 0.912
P(AH/FH) = 0.411
ΔΨ(AH/FH) = 0.675
FDR(AM/AH) = 0.0529
P(AM/AH) = 0.00367
ΔΨ(AM/AH) = −22.6
FDR(FM/FH) = 0.894
P(FM/FH) = 0.401
ΔΨ(FM/FH) = −6.97
F
Exon 20
chr1:228256651–228256926
FDR(M) = 0.462
P(M) = 0.124
ΔΨ(M) = −5.46
*FDR(A) = 0.0241
*P(A) = 0.00526
*ΔΨ(A) = −7.59
*FDR(AM/FM) = 0.0143
*P(AM/FM) = 0.00283
*ΔΨ(AM/FM) = −17.5
FDR(AH/FH) = 0.976
P(AH/FH) = 0.579
ΔΨ(AH/FH) = 2.28
FDR(AM/AH) = 0.325
P(AM/AH) = 0.0586
ΔΨ(AM/AH) = −15.3
FDR(FM/FH) = 0.998
P(FM/FH) = 0.856
ΔΨ(FM/FH) = 4.42
G
Exon 23
chr1:228268530–228268805
FDR(M) = 0.754
P(M) = 0.361
ΔΨ(M) = −1.16
*FDR(A) = 0.0203
*P(A) = 0.00425
*ΔΨ(A) = −7.36
*FDR(AM/FM) = 0.0118
*P(AM/FM) = 0.00223
*ΔΨ(AM/FM) = −14.4
FDR(AH/FH) = 0.783
P(AH/FH) = 0.265
ΔΨ(AH/FH) = −0.285
FDR(AM/AH) = 0.678
P(AM/AH) = 0.242
ΔΨ(AM/AH) = −8.24
FDR(FM/FH) = 0.991
P(FM/FH) = 0.752
ΔΨ(FM/FH) = 5.91
H
Exon 48
chr1:228291898–228292161
*FDR(M) = 3.03e−06
*P(M) = 4.25e−08
*ΔΨ(M) = 44.6
*FDR(A) = 0.000128
*P(A) = 1.12e−05
*ΔΨ(A) = −10.3
*FDR(AM/FM) = 1.92e−06
*P(AM/FM) = 1.03e−07
*ΔΨ(AM/FM) = −13
FDR(AH/FH) = 0.816
P(AH/FH) = 0.294
ΔΨ(AH/FH) = −7.65
*FDR(AM/AH) = 0.00374
*P(AM/AH) = 0.00012
*ΔΨ(AM/AH) = 41.9
*FDR(FM/FH) = 5.92e−08
*P(FM/FH) = 3.1e−10
*ΔΨ(FM/FH) = 47.2
I
Exon 49
chr1:228292523–228292786
*FDR(M) = 0.0292
*P(M) = 0.00188
*ΔΨ(M) = 37.2
*FDR(A) = 3.3e−06
*P(A) = 1.82e−07
*ΔΨ(A) = −12.7
*FDR(AM/FM) = 3.25e−08
*P(AM/FM) = 1.17e−09
*ΔΨ(AM/FM) = −23.4
FDR(AH/FH) = 0.973
P(AH/FH) = 0.567
ΔΨ(AH/FH) = −1.9
FDR(AM/AH) = 0.426
P(AM/AH) = 0.095
ΔΨ(AM/AH) = 26.4
*FDR(FM/FH) = 3.64e−06
*P(FM/FH) = 2.82e−08
*ΔΨ(FM/FH) = 47.9
J
Exon 50
chr1:228293353–228293616
FDR(M) = 0.126
P(M) = 0.0148
ΔΨ(M) = 30.2
*FDR(A) = 0.00784
*P(A) = 0.00135
*ΔΨ(A) = −10.4
*FDR(AM/FM) = 0.00388
*P(AM/FM) = 0.000592
*ΔΨ(AM/FM) = −13.9
FDR(AH/FH) = 0.673
P(AH/FH) = 0.183
ΔΨ(AH/FH) = −7
FDR(AM/AH) = 0.435
P(AM/AH) = 0.0985
ΔΨ(AM/AH) = 26.7
FDR(FM/FH) = 0.0945
P(FM/FH) = 0.00534
ΔΨ(FM/FH) = 33.6
K
Exon 51
chr1:228294152–228294415
FDR(M) = 0.276
P(M) = 0.0505
ΔΨ(M) = 26.7
*FDR(A) = 0.00385
*P(A) = 0.000575
*ΔΨ(A) = −12.6
*FDR(AM/FM) = 0.00235
*P(AM/FM) = 0.000328
*ΔΨ(AM/FM) = −14.9
FDR(AH/FH) = 0.471
P(AH/FH) = 0.0856
ΔΨ(AH/FH) = −10.3
FDR(AM/AH) = 0.61
P(AM/AH) = 0.192
ΔΨ(AM/AH) = 24.4
FDR(FM/FH) = 0.221
P(FM/FH) = 0.0209
ΔΨ(FM/FH) = 29
L
Exon 52
chr1:228294780–228295043
*FDR(M) = 0.0131
*P(M) = 0.000658
*ΔΨ(M) = 34.5
FDR(A) = 0.0547
P(A) = 0.0147
ΔΨ(A) = −5.07
*FDR(AM/FM) = 0.0266
*P(AM/FM) = 0.006
*ΔΨ(AM/FM) = −10.9
FDR(AH/FH) = 0.992
P(AH/FH) = 0.635
ΔΨ(AH/FH) = 0.741
FDR(AM/AH) = 0.181
P(AM/AH) = 0.0226
ΔΨ(AM/AH) = 28.7
*FDR(FM/FH) = 0.00633
*P(FM/FH) = 0.000143
*ΔΨ(FM/FH) = 40.3
M
Exon 53
chr1:228298454–228298717
*FDR(M) = 2.6e−06
*P(M) = 3.61e−08
*ΔΨ(M) = 48.5
*FDR(A) = 0.00286
*P(A) = 0.000404
*ΔΨ(A) = −10.6
*FDR(AM/FM) = 0.000443
*P(AM/FM) = 4.75e−05
*ΔΨ(AM/FM) = −17.7
FDR(AH/FH) = 0.79
P(AH/FH) = 0.27
ΔΨ(AH/FH) = −3.4
*FDR(AM/AH) = 0.00142
*P(AM/AH) = 3.73e−05
*ΔΨ(AM/AH) = 41.4
*FDR(FM/FH) = 1.46e−06
*P(FM/FH) = 1.03e−08
*ΔΨ(FM/FH) = 55.7
N
Exon 54
chr1:228299259–228299522
*FDR(M) = 5.26e−05
*P(M) = 9.68e−07
*ΔΨ(M) = 34.7
FDR(A) = 0.0538
P(A) = 0.0144
ΔΨ(A) = −7.82
*FDR(AM/FM) = 0.01
*P(AM/FM) = 0.00184
*ΔΨ(AM/FM) = −12.3
FDR(AH/FH) = 0.812
P(AH/FH) = 0.29
ΔΨ(AH/FH) = −3.34
*FDR(AM/AH) = 0.0101
*P(AM/AH) = 0.000412
*ΔΨ(AM/AH) = 30.2
*FDR(FM/FH) = 1.28e−05
*P(FM/FH) = 1.13e−07
*ΔΨ(FM/FH) = 39.2
O
Exon 55
chr1:228299882–228300145
*FDR(M) = 0.00387
*P(M) = 0.000141
*ΔΨ(M) = 37.1
*FDR(A) = 3.32e−06
*P(A) = 1.83e−07
*ΔΨ(A) = −17.3
*FDR(AM/FM) = 6.86e−09
*P(AM/FM) = 2.18e−10
*ΔΨ(AM/FM) = −29.2
FDR(AH/FH) = 0.699
P(AH/FH) = 0.2
ΔΨ(AH/FH) = −5.31
FDR(AM/AH) = 0.134
P(AM/AH) = 0.0141
ΔΨ(AM/AH) = 25.1
*FDR(FM/FH) = 1.62e−06
*P(FM/FH) = 1.16e−08
*ΔΨ(FM/FH) = 49
P
Exon 56
chr1:228303675–228303938
*FDR(M) = 5.28e−05
*P(M) = 9.72e−07
*ΔΨ(M) = 43.4
*FDR(A) = 0.0141
*P(A) = 0.00273
*ΔΨ(A) = −11.8
*FDR(AM/FM) = 0.00787
*P(AM/FM) = 0.00137
*ΔΨ(AM/FM) = −11.1
FDR(AH/FH) = 0.107
P(AH/FH) = 0.00659
ΔΨ(AH/FH) = −12.5
*FDR(AM/AH) = 0.00179
*P(AM/AH) = 4.88e−05
*ΔΨ(AM/AH) = 44.1
*FDR(FM/FH) = 0.00216
*P(FM/FH) = 3.92e−05
*ΔΨ(FM/FH) = 42.7
Q
Exon 57
chr1:228304303–228304566
*FDR(M) = 3.36e−08
*P(M) = 3.49e−10
*ΔΨ(M) = 52.1
*FDR(A) = 0.00465
*P(A) = 0.000721
*ΔΨ(A) = −12.4
*FDR(AM/FM) = 0.0013
*P(AM/FM) = 0.000165
*ΔΨ(AM/FM) = −9.74
FDR(AH/FH) = 0.141
P(AH/FH) = 0.0104
ΔΨ(AH/FH) = −15.1
*FDR(AM/AH) = 7.79e−06
*P(AM/AH) = 9.13e−08
*ΔΨ(AM/AH) = 54.7
*FDR(FM/FH) = 5.06e−07
*P(FM/FH) = 3.23e−09
*ΔΨ(FM/FH) = 49.4
R
Exon 58
chr1:228305123–228305386
*FDR(M) = 5.15e−14
*P(M) = 2.97e−16
*ΔΨ(M) = 53.2
FDR(A) = 0.147
P(A) = 0.0538
ΔΨ(A) = −8.41
FDR(AM/FM) = 0.108
P(AM/FM) = 0.0359
ΔΨ(AM/FM) = −3.02
FDR(AH/FH) = 0.266
P(AH/FH) = 0.0302
ΔΨ(AH/FH) = −13.8
*FDR(AM/AH) = 1.09e−08
*P(AM/AH) = 7.46e−11
*ΔΨ(AM/AH) = 58.6
*FDR(FM/FH) = 7.08e−09
*P(FM/FH) = 3.04e−11
*ΔΨ(FM/FH) = 47.8
S
Exon 97
chr1:228356151–228356193
*FDR(M) = 0.0123
*P(M) = 0.000602
*ΔΨ(M) = −16.1
*FDR(A) = 1.1e−22
*P(A) = 4.51e−25
*ΔΨ(A) = −24.6
*FDR(AM/FM) = 4.36e−28
*P(AM/FM) = 1.33e−30
*ΔΨ(AM/FM) = −44.1
FDR(AH/FH) = 0.141
P(AH/FH) = 0.0104
ΔΨ(AH/FH) = −5.13
*FDR(AM/AH) = 0.000144
*P(AM/AH) = 2.52e−06
*ΔΨ(AM/AH) = −35.6
FDR(FM/FH) = 0.95
P(FM/FH) = 0.53
ΔΨ(FM/FH) = 3.35
T
Exon 107
chr1:228368339–228368416
FDR(M) = 0.942
P(M) = 0.746
ΔΨ(M) = −5.69
*FDR(A) = 4.05e−15
*P(A) = 4.11e−17
*ΔΨ(A) = 21.2
*FDR(AM/FM) = 2.44e−16
*P(AM/FM) = 2.54e−18
*ΔΨ(AM/FM) = 27.4
*FDR(AH/FH) = 0.0247
*P(AH/FH) = 0.000715
*ΔΨ(AH/FH) = 15.1
FDR(AM/AH) = 0.981
P(AM/AH) = 0.862
ΔΨ(AM/AH) = 0.477
FDR(FM/FH) = 0.603
P(FM/FH) = 0.139
ΔΨ(FM/FH) = −11.9
U
Exon 111
chr1:228370193–228370238
*FDR(M) = 1.65e−07
*P(M) = 1.88e−09
*ΔΨ(M) = 9.41
*FDR(A) = 0.00525
*P(A) = 0.000832
*ΔΨ(A) = 4.11
FDR(AM/FM) = 0.212
P(AM/FM) = 0.0883
ΔΨ(AM/FM) = 2.47
FDR(AH/FH) = 0.948
P(AH/FH) = 0.492
ΔΨ(AH/FH) = 5.74
*FDR(AM/AH) = 2.29e−05
*P(AM/AH) = 3.05e−07
*ΔΨ(AM/AH) = 7.78
*FDR(FM/FH) = 0.00121
*P(FM/FH) = 2.02e−05
*ΔΨ(FM/FH) = 11

Figure S3. Inclusion levels of the studied exons of *OBSCN*: Boxplots illustrating the distribution of the Ψ levels of the studied *OBSCN* exons. The sample classes for which the Ψ values are shown are: postnatal muscles (AM), postnatal hearts (AH), fetal muscles (FM), and fetal hearts (FH). The FDR, P-value and $\Delta\Psi$ values for all the comparisons are listed below the box plots. The significant results *(i.e.* FDR < 0.05) are marked with * and coloured in red. Furthermore, the P-values and the FDR values of the first and last exons are shown with NA as no RNA-Seq reads skip these exons. The value for exon 126 is omitted, as its differences from exon 125 are minute (*i.e.* it extends on the 3' end by only 2 nucleotides). The comparisons include: muscle vs heart (M), postnatal vs fetal (A), postnatal muscle vs other samples (AM), postnatal heart vs other samples (AH), fetal muscle vs other samples (EM), and fetal heart vs other samples (FH). The box plots extend from the 25th to the 75th percentile, and the thick horizontal line represents the median. The boxplot whiskers show 1.5 times the interquartile range. The outliers are values higher and lower than the interquartile range.

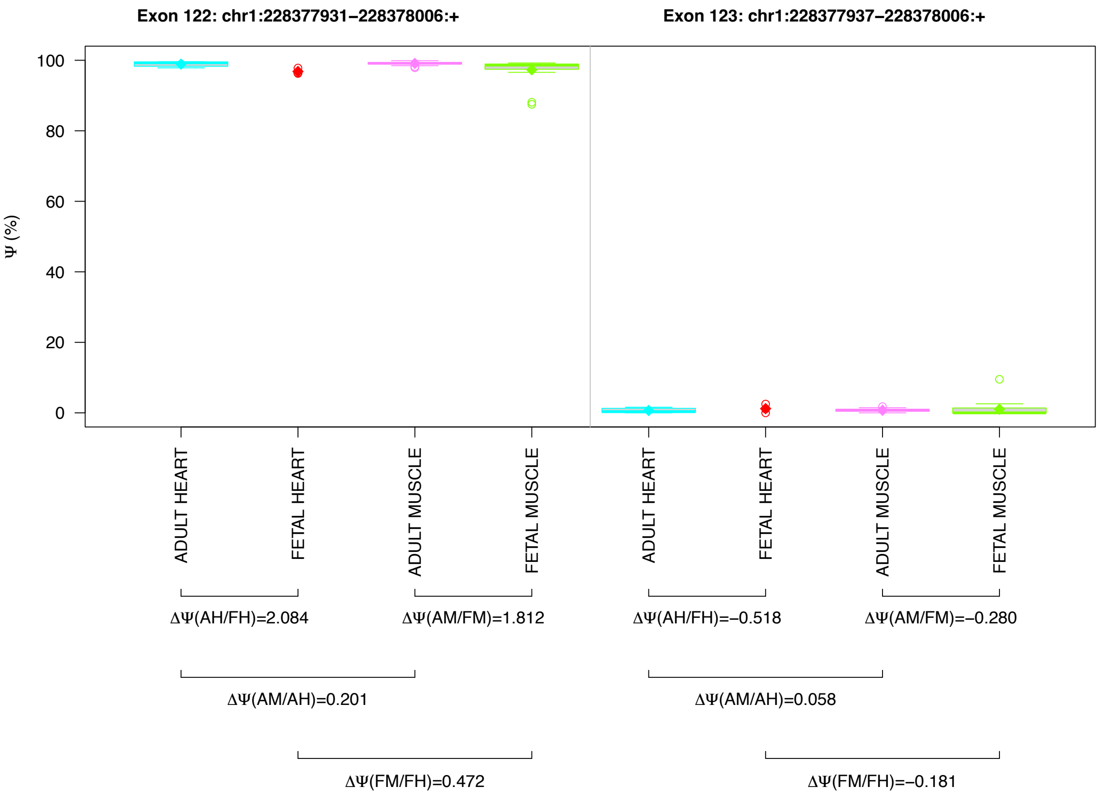

Figure S4. Alternative 3' splicing: Boxplots illustrating the distribution of the Ψ levels of the alternative 3' splicing. The headers show the coordinates of the affected exons. The sample classes for which the Ψ values are shown are: postnatal muscles (AM), postnatal hearts (AH), fetal muscles (FM), and fetal hearts (FH). The $\Delta\Psi$ values for two sets of comparisons are listed below the box plots: postnatal heart vs postnatal muscle (AH/AM), and fetal heart vs fetal muscle (EH/EM). The box plots extend from the 25th to the 75th percentile, and the thick horizontal line represents the median. The boxplot whiskers show 1.5 times the interquartile range. The outliers are values higher and lower than the interquartile range.
